# Immediate myeloid depot for SARS-CoV-2 in the human lung

**DOI:** 10.1126/sciadv.adm8836

**Published:** 2024-07-31

**Authors:** Mélia Magnen, Ran You, Arjun A. Rao, Ryan T. Davis, Lauren Rodriguez, Olivier Bernard, Camille R. Simoneau, Lisiena Hysenaj, Kenneth H. Hu, Mazharul Maishan, Catharina Conrad, Oghenekevwe M. Gbenedio, Bushra Samad, The UCSF COMET Consortium, Christina Love, Prescott G. Woodruff, David J. Erle, Carolyn M. Hendrickson, Carolyn S. Calfee, Michael A. Matthay, Jeroen P. Roose, Anita Sil, Melanie Ott, Charles R. Langelier, Matthew F. Krummel, Mark R. Looney

**Affiliations:** ^1^ Department of Medicine, University of California, San Francisco, San Francisco, CA 94143, USA.; ^2^ Department of Pathology, University of California, San Francisco, San Francisco, CA 94143, USA.; ^3^ CoLabs Initiative, University of California, San Francisco, San Francisco, CA 94143, USA.; ^4^ Department of Microbiology and Immunology, University of California, San Francisco, San Francisco, CA 94143, USA.; ^5^ Gladstone Institutes, San Francisco, CA 94158, USA.; ^6^ Department of Anatomy, University of California, San Francisco, San Francisco, CA 94143, USA.; ^7^ All UCSF COMET Consortium collaborators are affiliated with the University of California, San Francisco, San Francisco, CA 94143, USA.; ^8^ Chan Zuckerberg Biohub, San Francisco, CA 94158, USA.

## Abstract

In the pathogenesis of severe acute respiratory syndrome coronavirus 2 (SARS-CoV-2) infection, epithelial populations in the distal lung expressing Angiotensin-converting enzyme 2 (ACE2) are infrequent, and therefore, the model of viral expansion and immune cell engagement remains incompletely understood. Using human lungs to investigate early host-viral pathogenesis, we found that SARS-CoV-2 had a rapid and specific tropism for myeloid populations. Human alveolar macrophages (AMs) reliably expressed ACE2 allowing both spike-ACE2–dependent viral entry and infection. In contrast to Influenza A virus, SARS-CoV-2 infection of AMs was productive, amplifying viral titers. While AMs generated new viruses, the interferon responses to SARS-CoV-2 were muted, hiding the viral dissemination from specific antiviral immune responses. The reliable and veiled viral depot in myeloid cells in the very early phases of SARS-CoV-2 infection of human lungs enables viral expansion in the distal lung and potentially licenses subsequent immune pathologies.

## Teaser

Alveolar macrophages are a depot for SARS-CoV-2 leading to productive infection with minimal immune stimulating cues.

## INTRODUCTION

The severe acute respiratory syndrome coronavirus 2 (SARS-CoV-2) pandemic has led to millions of deaths worldwide. During natural infection, SARS-CoV-2 is known to infect ciliated cells in the nasopharynx or trachea. In serious cases, the virus is not cleared by the immune system leading to spread along the tracheobronchial tree into the lower respiratory tract (*1*). However, in the distal lung, only a small subset of epithelial cells expresses the main SARS-CoV-2 entry receptor—ACE2 (*2*, *3*). This suggests the involvement of other cell(s) in the lung, including potentially immune cells, in the expansion of virus in the distal lung. This very early pathogenesis of COVID-19 is completely unknown.

The access to large numbers of human patient samples has enabled extensive studies of the immune responses to COVID-19 (*4*–*6*). Unfortunately, once patients are hospitalized, the host-pathogen responses have been in progress for days or weeks, creating a challenging problem for understanding the very early host responses to SARS-CoV-2 infection in humans. Monocytes are infected in such late responses, partly mediated by antibodies (*7*). On the converse, animal models or human lung organoids are mute in being able to reliably report the nature of infection in the replete setting of the human lung. Here, we used precision-cut lung slices (PCLSs) of human lungs to study early host-pathogen responses in a system with the full repertoire of lung stromal and immune cells. We infected the PCLSs with SARS-CoV-2 and compared responses to another pandemic virus—Influenza A. The results unambiguously demonstrate an immediate host tropism of SARS-CoV-2 for human alveolar macrophages (AMs)—an ontologically distinct immune cell population in the lung (*8*). The significance of this work is that in the early phases of SARS-CoV-2 infection, direct infection of the immune system may enhance viral dissemination and enable subsequent immune pathology, such as the acute respiratory distress syndrome (ARDS)—the principal cause of death in severe COVID-19.

## RESULTS

### Epithelial and immune cell populations are infected by SARS-CoV-2 in human lung slices

**Fig. 1.**
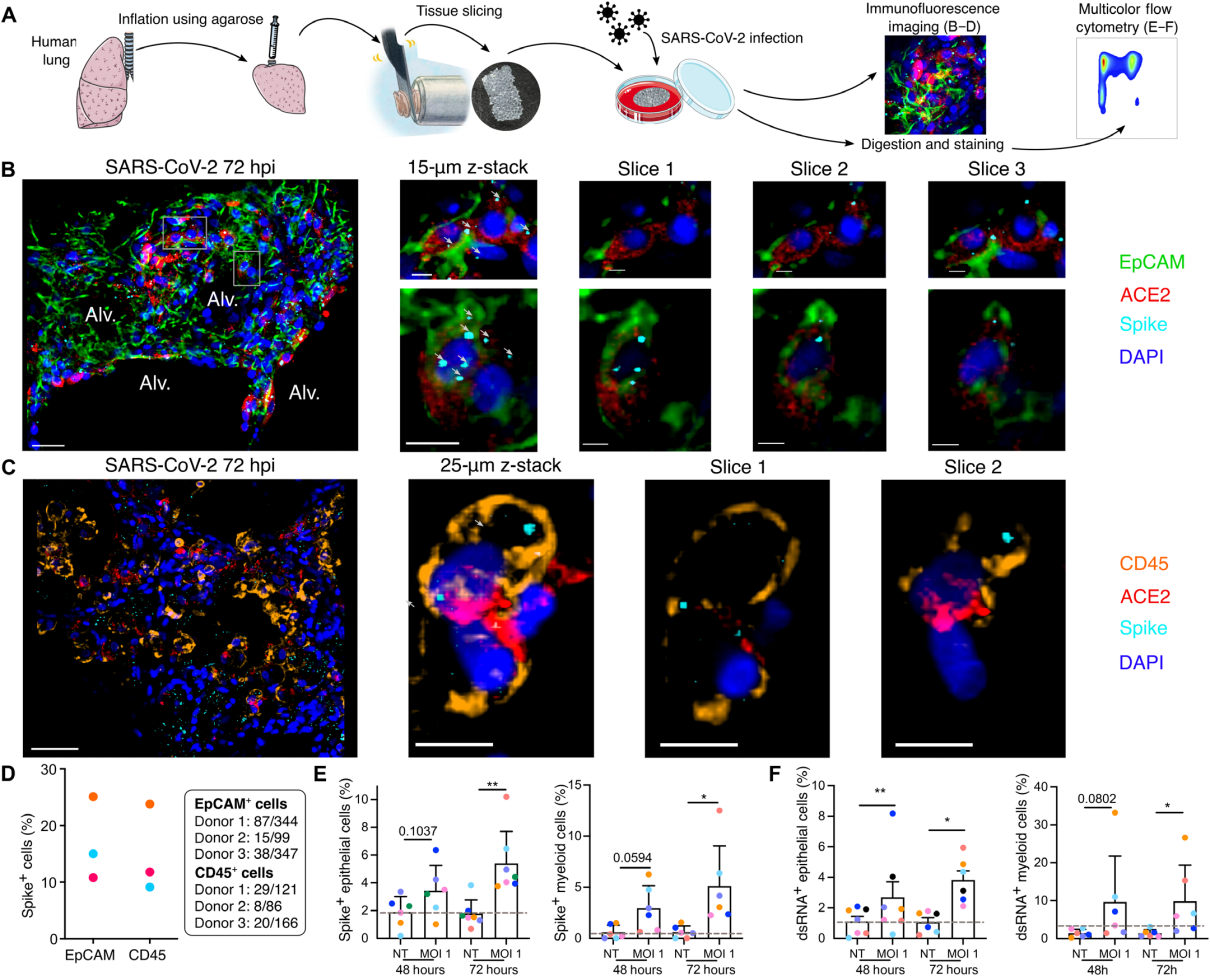
SARS-CoV-2 infects both epithelial and immune cells in human PCLS. (**A**) Human lung lobes were inflated with 2% low–melting point agarose and sectioned into 300-μm PCLSs, which were infected with SARS-CoV-2 for 48 or 72 hours. PCLS were either used for confocal imaging (B to D) or flow cytometry (E and F). (**B** and **C**) Alveolar spaces (Alv.) are indicated in the large image (scale bar, 50 μm). Zoomed area is marked by the white rectangle. For each zoomed area, 15- or 25-μm z-stacks appear on the side (scale bar, 10 μm). (B) PCLS were stained for 4′,6-diamidino-2-phenylindole (DAPI) (dark blue), EpCAM (green), ACE2 (red), and spike (light blue). (C) PCLS were stained for DAPI (dark blue), CD45 (orange), ACE2 (red), and spike (light blue). (**D**) For both stainings, spike-positive cell percentages are displayed for three donors. (**E** and **F**) PCLS were infected at MOI 1. Infection was assessed by intracellular spike and dsRNA staining in epithelial (E) and myeloid cells (F) (*n* = 6 to 7). Gray dashed lines indicate detection limit of assays. Data are means ± SEM. Each dot represents the average percentage per donor of two to three PCLS. *P < 0.05, **P < 0.01.

We have previously developed a model of PCLS in the mouse lung (*9*, *10*) that we have now applied to human lungs donated for research purposes (table S1). A lung lobe was inflated using 2% low–melting point agarose, and 300-μm PCLS were produced for tissue culture and direct viral infection (Fig. 1A). Infection with SARS-CoV-2 [USA-WA1/2020; multiplicity of infection (MOI) 0.1] or Influenza A virus (IAV; MOI 0.1) both produced increasing viral titers in the PCLS at 48 and 72 hours (fig. S1, A and B). We first focused on the specific populations of lung cells that were infected by SARS-CoV-2. Imaging of the infected PCLS (Fig. 1, B to D; noninfected in figs. S1 and S2) revealed spike staining in epithelial cells [epithelial cell adhesion molecule–positive (EpCAM^+^)] that were also ACE2 positive (Fig. 1B and fig. S1C). To further investigate infection, PCLSs were enzymatically digested for multicolor flow cytometry analysis (Fig. 1A). Cells were stained for spike and double-stranded RNA (dsRNA) (Fig. 1, E and F; gating strategy in fig. S3, B and D). Epithelial cells (CD45^−^ EpCAM^+^ CD31^−^) displayed increasing spike and dsRNA signal after 72 hours of SARS-CoV-2 infection (Fig. 1, E and F, and fig. S2A). Both imaging and flow cytometry showed that SARS-CoV-2 was able to induce epithelial infection in a small-scale human lung model.

However, using both imaging and flow cytometry, we also observed spike and dsRNA signal in lung immune cells (Fig. 1, C, E, and F), the former prevalent from the earliest 48-hour time point. Spike was colocalized to CD45^+^ ACE2^+^ cells (Fig. 1C; noninfected in fig. S1D), and similarly, dsRNA was found in these cells (fig. S2B), supporting that concept that immune cells may either be infected by SARS-CoV-2 or phagocytose the virus. Quantification of the immunofluorescence imaging revealed that proportions of spike^+^ cells among the EpCAM^+^ and CD45^+^ population were similar (Fig. 1D). Further analysis of ACE2 staining showed that the majority of spike^+^ CD45^+^ cells were ACE2^+^ (fig. S3C), similar to EpCAM^+^ cells (fig. S3A). Flow cytometry allowed us to further characterize spike^+^ and dsRNA^+^ immune cells, the latter considered indicative of ongoing viral replication (Fig. 1, E and F). We observed significant spike and dsRNA signal in lung myeloid cells [CD45^+^ CD3^−^ CD19^−^ HLA-DR^+^ CD14^+^ cells, including macrophages, monocytes, and monocyte-derived dendritic cells (DCs); (*11*)] at 72 hours after infection (Fig. 1, E and F). An important caveat for interpreting these data is the unique susceptibility of epithelial cell populations to enzymatic digestion, which may limit recovery. Overall, these data support that immune cells, particularly myeloid cells, have unique and early interactions with SARS-CoV-2, potentially leading to a major role in shaping the immune response.

### Single-cell RNA sequencing reveals myeloid tropism of SARS-CoV-2 in the human lung

**Fig. 2.**
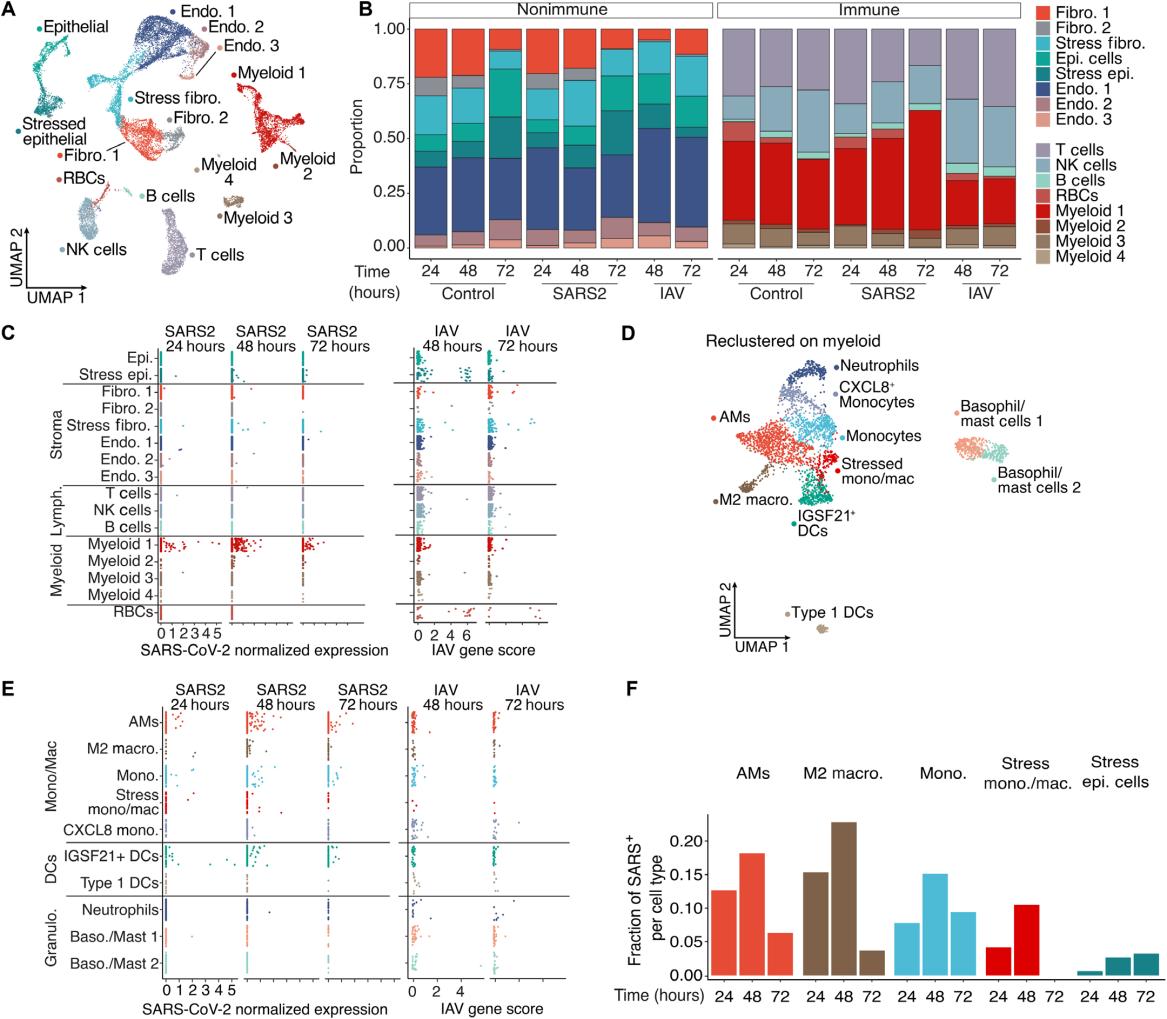
SARS-CoV-2 displays tropism for myeloid cells compared to IAV. (**A**) A Uniform Manifold Approximation and Projection (UMAP) visualization of cells from control, SARS-CoV-2, and IAV-infected PCLS (two donors, three technical replicates) collected at distinct times. (**B**) Relative quantification of cell types from the different experimental conditions, stratified by time point. (**C**) Scatterplots describing the library-normalized SARS-CoV-2 expression across various cell types in SARS-CoV-2–infected PCLS (left) or IAV gene score in IAV-infected PCLS (right). (**D**) A UMAP of finely annotated myeloid cell types in the dataset. (**E**) Distribution of infected myeloid cells similar to (C). (**F**) The fraction of SARS-CoV-2–positive cells at different time points.

To further characterize the transcriptional influence of SARS-CoV-2 infection on the entire contents of the human lung during the first days of exposure, we applied single-cell RNA sequencing (scRNA-seq) analysis to cells obtained at various time points after PCLS infection (Fig. 2). To appreciate unique changes in lung composition and gene expression induced by SARS-CoV-2, we compared lung slices infected by either SARS-CoV-2 or IAV. Hierarchical analysis identified the anticipated clusters representing highly heterogeneous lung complexity, including eight populations of nonimmune cells, lymphocytes (T cells, B cells, and natural killer cells), and four populations of myeloid cells (Fig. 2A and fig. S4A) (*12*). In response to IAV, the most profound change was a reduction in lung fibroblast (48 hours) and epithelial cell (72 hours) proportions, consistent with previous reports (*13*) and more modest reductions in myeloid cells (Fig. 2B and fig. S4A). SARS-CoV-2, in contrast, produced no notable trends in lung nonimmune cell populations, compared to controls and led to an almost 50% increase in the myeloid fraction over time relative to controls at 72 hours (Fig. 2B).

We next tracked viral infection cell-by-cell by aligning the scRNA-seq data on the two viral genomes. This revealed that the main targets for IAV infection were epithelial cells and fibroblasts (Fig. 2C), consistent with the observed loss of these populations in PCLS (Fig. 2B). In addition, IAV reads were also sporadically distributed in several other populations including immune cells. This may be attributable to the IAV entry receptor (sialic acid) being widely distributed on the surfaces of many cell types or perhaps to phagocytosis. SARS-CoV-2 reads by comparison were distinctly enriched in myeloid cells, even at the earliest 24-hour time point. Only a few reads were identified in nonimmune cells (Fig. 2C). This may relate to overall reduced cellular recovery/encapsulation of some lung stromal cells with scRNA-seq.

Delving into the subpopulation of myeloid cells targeted by SARS-CoV-2, we recombined and reclustered the four myeloid clusters from Fig. 2A, resulting in definition of 10 myeloid populations that included neutrophils, DCs, and multiple subpopulations of monocytes and macrophages (Fig. 2D and fig. S4B), consistent with a previous lung atlas (*12*). IAV reads remained low and sporadic across all 10 clusters (Fig. 2E). Conversely, SARS-CoV-2 tropism was more selective, with AMs, IGSF21^+^ DCs, and monocytes accounting for the majority of viral reads (Fig. 2E). To account for the variable frequency of each population in Fig. 2E, we analyzed the SARS-CoV-2 read frequency and plotted this over time. This demonstrated that viral reads rose in unison in both myeloid and epithelial cell populations from 24 to 48 hours and then decreased in the myeloid populations at 72 hours (Fig. 2F) perhaps from resolution of the infection in the PCLS or an exhaustion of host cells targeted by the virus.

### SARS-CoV-2 displays a myeloid tropism in human endotracheal aspirates from COVID-19 ARDS

**Fig. 3.**
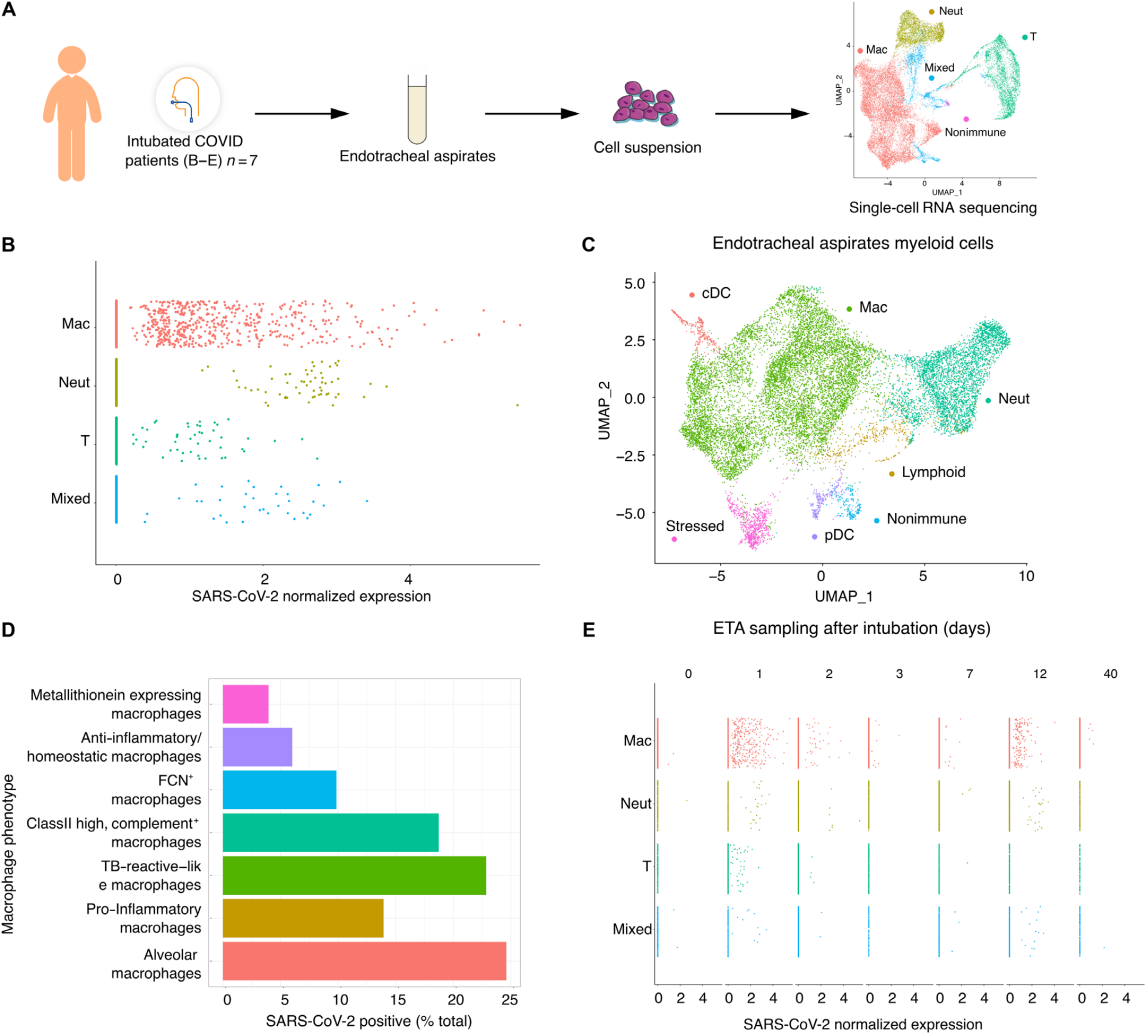
Tropism of SARS-CoV-2 for myeloid cells in ETAs from COVID-19 individuals with ARDS. (**A**) ETAs were collected from SARS-CoV-2 infected individuals with ARDS and tested with scRNA-seq. UMAP at far right shows landmark populations. (**B**) Distribution of per-cell normalized SARS-CoV-2 expression in landmark cell types. (**C**) UMAP projection of myeloid subtypes in ETAs. (**D**) Fraction of SARS-CoV-2–positive cells per myeloid cell type (**E**) Days (D0 to D40) at the top of the graph represent the days after intubation when the ETA samples were collected. Each column represents a unique COVID-19 individual except for postintubation day 2, where two individuals are represented. See table S2 for details.

To compare our findings in the isolated human lung model to clinical COVID-19 cases, we sampled endotracheal aspirates (ETAs) from seven intubated individuals with ARDS from COVID-19 (table S2) and analyzed by scRNA-seq (Fig. 3). Clustering showed that the cellular population was predominantly composed of myeloid cells (Fig. 3A, far right), which contained macrophages, neutrophils, and some DCs (Fig. 3C). Analysis of SARS-CoV-2 normalized expression revealed that SARS-CoV-2 reads localized mainly to macrophages but in some cases were also found in neutrophils and T cells (Fig. 3B), similar to prior results (*14*). The ETA samples were obtained at different times after intubation, but the timing did not correlate with the quantity or SARS-CoV-2 reads (Fig. 3E). One individual was sampled at 40 days after intubation but still had detectable viral reads in macrophages, which may point to a depot effect that can be quite long lasting. As in the PCLS model, several macrophage subpopulations (fig. S5) were found to have SARS-CoV-2 reads (Fig. 3D). The most notable example was AMs with almost 25% being viral RNA positive.

### AMs express ACE2 RNA and protein

**Fig. 4.**
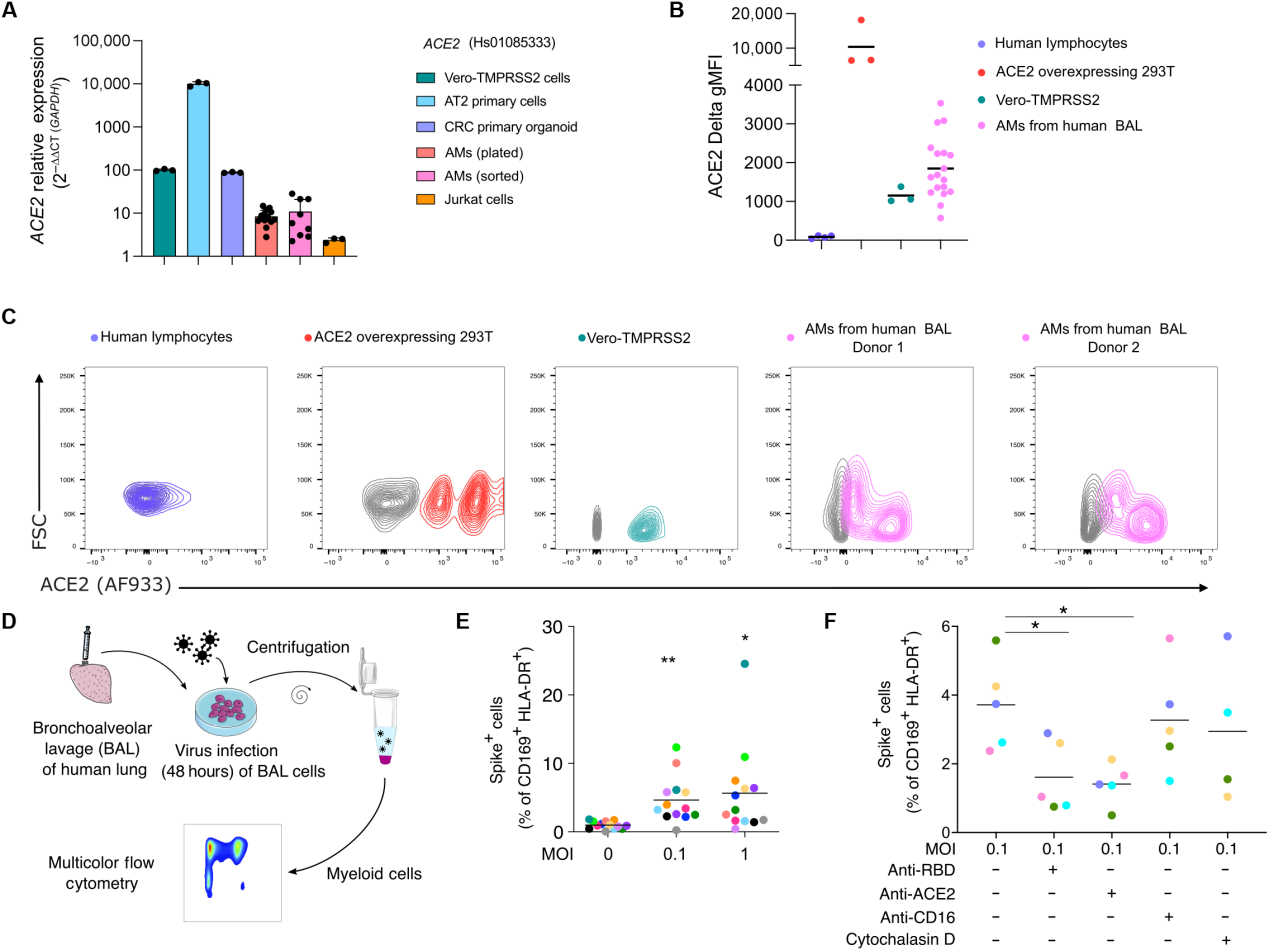
SARS-CoV-2 infection of AMs is ACE2 dependent. (**A** to **C**) ACE2 mRNA and protein expression was assessed on AMs (AMs: CD169^+^ HLA-DR^+^ cells) from BAL of human lungs. (A) *ACE2* transcript expression was assessed by RT-qPCR in Vero-TMPRSS2 cells (green), AT2 primary cells (blue), colorectal cancer (CRC) organoid (violet), Jurkat cells (orange), and AMs from human BAL [red (plated) and pink (flow-sorted), *n* = 3 donors]. Relative mRNA expression is displayed as 2^−∆∆CT^ relative to *GAPDH* for probe Hs1085333. Vero-TMPRSS2 are set at 100. Bars show means ± SD. (B) and (C) ACE2 protein expression was measured in AMs from human BAL (*n* = 18, each dot is a donor), in Vero-TMPRSS2 cells (*n* = 3), in ACE2-overexpressing 293 T cells (ACE2-293 T, *n* = 3), and in human lymphocytes from PBMCs (*n* = 4). Delta MFI (Stained MFI − FMO MFI) was measured for each population. Results are displayed as contour plots, gray lines represent FMO staining, and colored lines are the fully stained samples. (**D** and **E**) AM infection (MOI 0.1 and 1) was measured by flow cytometry using spike staining and compared to nontreated (MOI 0) cells (mean, each color represents a human lung donor, *n* = 13). (**F**) RBD, ACE2, or CD16 blocking antibody or cytochalasin D was added to BAL cells before infection (MOI 0.1). Cells were analyzed by flow cytometry (*n* = 4 to 5). *P < 0.05, **P < 0.01.

To investigate how SARS-CoV-2 affects human lung myeloid cells, we focused on the dominant AMs, which are also anatomically within the airspaces and so directly exposed to virus (*15*). Published studies on human AMs have notably failed to detect *ACE2* mRNA by scRNA-seq (table S3). However, that method has low read depths, and multiple studies have found ACE2 using protein detection methods (table S3). Our scRNA-seq data were consistent with previous studies, failing to detect *ACE2* RNA in both SARS-CoV-2 infected and uninfected PCLSs and could only detect *ACE2* in modest numbers of stressed fibroblasts, epithelial cells, and stressed epithelial cells but not myeloid cells (fig. S6A). Reasoning that scRNA-seq may not be sensitive for transcripts of lower abundance (*16*), we used real-time quantitative polymerase chain reaction (RT-qPCR) to measure *ACE2* mRNA expression in plated and sorted AMs (Live, CD45^+^ CD169^+^ HLA-DR^+^, CD3^−^, CD19^−^, and EpCAM^−^) obtained by bronchoalveolar lavage (BAL) using two primer sets. Our controls, primary alveolar epithelial type II (ATII) cells (*17*), colorectal carcinoma (CRC) organoids (*18*), and Vero-TMPRSS2 cells, all expressed various levels of *ACE2* mRNA with ATII cells having the most expression (Fig. 4A and fig. S6, B and C). Plated and sorted AMs (three donors) had lower levels of *ACE2* mRNA versus Vero-TMPRSS2 and CRC organoids but higher levels than Jurkat cells (T lymphocytes) using both primer sets (Fig. 4A and fig. S6, B and C).

We also tested for ACE2 protein expression using flow cytometry and Western blotting. For these experiments, we used BAL cells enriched in AMs, defined here as CD45^+^ CD169^+^ HLA-DR^+^ (fig. S7, A and B) (*19*) and comprising very few epithelial cells (<1% of live cells) (fig. S7, C and D). Flow cytometry using four different antibodies showed that AMs (18 donors) expressed ACE2 (Fig. 4, B and C, and fig. S8, A to C), and in many, we noted AMs with two or more levels of surface ACE2, which might be a result of differential display or perhaps internalization following ligand exposure. ACE2 overexpressing 293 T cells and Vero-TMPRSS2 cells were used as positive controls and human lymphocytes as a negative control to validate antibody specificity. To further investigate ACE2 protein expression, sorted AMs from four BAL samples were used for Western blot analysis using two monoclonal antibodies (SN0754 and AC18F), confirming that AMs express ACE2 protein of the expected molecular weight (fig. S8, D and E).

### SARS-CoV-2 infects AMs via spike-ACE2 interactions

To study the direct interactions between AMs and SARS-CoV-2, we incubated the BAL cell population with SARS-CoV-2 and then analyzed cells by flow cytometry (Fig. 4, D to F, and fig. S9). After a 48-hour incubation with SARS-CoV-2 at MOI 0.1, we detected spike in up to 10% of AMs (Fig. 4E and fig. S9A). Somewhat unexpectedly, MOI 1 did not significantly increase the spike^+^ AM percentage compared to MOI 0.1 (Fig. 4E and fig. S9A), suggesting that a plateau was reached in the number of cells that were capable of being infected. The viability of AMs was high in both MOI groups (fig. S9B). Furthermore, the number of AMs recovered did not decrease in either MOI 0.1 or 1 infected conditions compared to uninfected control at 48 and 96 hours (fig. S9C), and there was no increase in apoptosis (annexin V) compared to uninfected cells (fig. S9D). In comparison, IAV showed both infection (fig. S9E) and increased apoptosis (fig. S9F) in AMs. Overall, our findings fail to show that SARS-CoV-2 induces cell death in AMs, contrary to observations in blood monocytes from COVID-19 individuals (*7*).

To investigate ACE2 mechanisms of viral entry into AMs, we used blocking antibodies targeting ACE2 (*20*) as well as the spike receptor binding domain (RBD). AMs were treated 2 hours before SARS-CoV-2 infection. Both approaches led to significant decreases in spike^+^ AMs (Fig. 4F and fig. S9G). Alternative routes of infection of myeloid cells include phagocytosis or antibody-mediated entry (CD16) (*7*). To test these mechanisms of virus entry, we used a CD16 neutralizing antibody and cytochalasin D (actin polymerization inhibitor). Both treatments did not significantly affect infection rate (Fig. 4F). Together, these data indicate that SARS-CoV-2 entry into AMs was substantially mediated by ACE2/spike RBD interactions.

### SARS-CoV-2 induces viral production by AMs

**Fig. 5.**
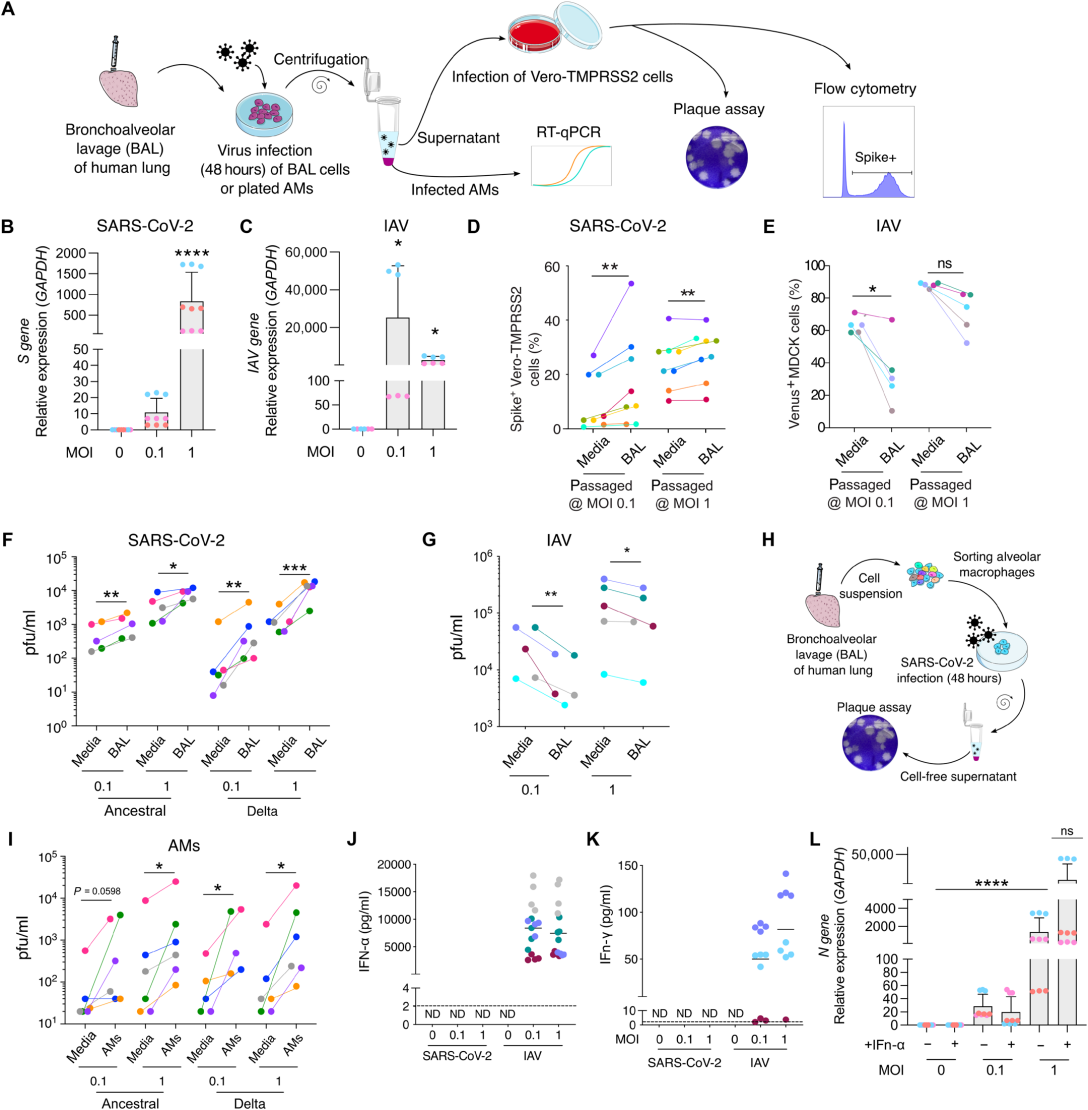
SARS-CoV-2 infection of AMs leads to viral amplification. (**A** to **C**) Virus gene expression was measured in plated AMs at 48 hours after infection with SARS-CoV-2 (B) or IAV (C). (A), (D), and (E) BAL cells were infected with SARS-CoV-2 (BAL). As a control, SARS-CoV-2 was incubated in culture media alone (Media). Cell-free supernatant was used to infect Vero-TMPRSS2 cells. (**D**) At 24 hours after infection, Vero-TMPRSS2 cells were stained for intracellular spike expression and analyzed using flow cytometry (*n* = 8). (**E**) Similarly, IAV-Venus was used to infect BAL cells or incubated with media. Cell-free supernatant was used to infect MDCK cells. At 24 hours after infection, Venus expressing MDCK percentage was measured by flow cytometry (*n* = 5). (**F** and **G**) Plaque assay was used to further assess viral titer in supernatant of infected BAL cells (*n* = 5 to 6). (**H**) AMs were sorted from BAL samples. (**I**) Following 48 hours of SARS-CoV-2 infection (Ancestral, Delta), viral titer was determined by plaque assay (*n* = 5 to 6). (**J** and **K**) BAL cells were infected for 48 hours with either IAV or SARS-CoV-2 at MOl 0. 1 or 1. After 48 hours of infection, IFN-α (J) and IFN-γ (K) were measured in the supernatant. (**L**) Plated AMs were infected with SARS-CoV-2 and treated with IFN-α (10 pg/ml) at 24 hours after infection. At 48 hours after infection, SARS-CoV-2 N gene expression was measured by RT-qPCR. ND, not detectable; ns, not significant; *P < 0.05, **P < 0.01, ***P < 0.001, ****P < 0.0001.

SARS-CoV-2 and IAV both infect AMs in our system (Fig. 5, A to C), but it is unknown whether SARS-CoV-2 can lead to productive infection. To study the ability of AMs that were exposed to virus to produce and release new viruses, SARS-CoV-2 and IAV (fluorescent strain, PR8-Venus) were incubated with BAL cells (MOI 0.1 or 1) as above, and the resulting titer in the media was then quantified. As a control, the same quantities of viruses were incubated with cell-free media. After 48 hours of incubation, cell-free supernatant was collected from the control (media) and BAL groups and used to infect Vero-TMPRSS2 (SARS-CoV-2) or Madin-Darby canine kidney (MDCK) cells (IAV). After 24 hours, the infection of these readout cells was assessed by spike staining and flow cytometry quantifying cells that were positive for spike (SARS-CoV-2) or for Venus (IAV) (Fig. 5A). For SARS-CoV-2, incubating the virus with BAL cells amplified virus at both MOI 0.1 and 1 compared to incubating with media alone (Fig. 5D, fold change in fig. S10A). We observed the opposite effect with IAV, in which the amount of virus decreased at both MOI 0.1 and 1 (Fig. 5E, fold change in fig. S10B).

In addition, we harvested supernatant for use in the gold-standard plaque assay (Fig. 5, A and F). BAL cells produced increased viral titers for both ancestral and delta (Fig. 5F, plaque assay in fig. S10C, fold change in fig. S10D) confirming productive infection of BAL cells by these variants. Again, IAV cultured with BAL led to opposite effects, namely, decreased viral titer in supernatants (Fig. 5G, fold change in fig. S10E).

Next, we purified AMs from BAL (flow-sorting gated as CD3^−^ CD19^−^ EpCAM^−^ CD45^+^ HLA-DR^+^ CD169^+^) before infecting them for 48 hours with SARS-CoV-2 variants (ancestral and delta) at MOI 0.1 or 1 (Fig. 5H). Consistent with results from BAL incubations, SARS-CoV-2 incubation with purified AMs induced an increase of viral titer in the resulting cell-free supernatant at both MOI 0.1 and 1 (Fig. 5I, plaque assay in fig. S10F, fold change in fig. S10G). This does not preclude that other populations in BAL might also be infected but clearly indicates AMs as being competent for viral replication.

### Antiviral responses are diminished in SARS-CoV-2–infected AMs compared to IAV

We examined the antiviral responses in AMs following IAV and SARS-CoV-2 exposure. Returning to the PCLS system and scRNA-seq readouts, we examined differentially expressed genes in AMs from PCLSs ± infection (fig. S11, A to C). IAV-infected PCLSs produced dominant increases in cytokine and interferon (IFN) signaling (fig. S11A), whereas these responses were absent in SARS-CoV-2 infection (fig. S11B). We next compared AMs with SARS-CoV-2 reads (infected) versus uninfected AMs (both from PCLSs) and found a broad up-regulation of immune responses (fig. S11C). Notably, apoptotic pathways were down-regulated (fig. S11C) consistent with our results on AM viability in fig. S9. We did find evidence that AMs exposed to SARS-CoV-2 had up-regulated IFN-stimulated genes (ISGs), but this induction was typically ~10-fold less in SARS-CoV-2, compared to IAV infection (fig. S11D). When we measured IFN levels in the supernatant of BAL cells at 48 hours after virus infection, we found that IAV triggered both IFN-α and IFN-γ release, but SARS-CoV-2 infection did not result in detectable levels of these antiviral proteins (Fig. 5, J and K). These findings were mirrored by both *IFNa* and *IFNg* gene expression analysis (fig. S11, E and F) in AMs isolated from BAL. We further confirmed that SARS-CoV-2–infected AMs (gated from BAL cells) displayed a modest increased level of one specific ISG, namely, IFITM3. This was only significant at high MOI of ancestral SARS-CoV-2 (fig. S11, G and H); conversely, IAV induced robust increases in gene expression at both low and high MOI (fig. S11H). Others ISGs, (*ISG15*, *IFI16*, and *IRF7*) were not increased following SARS-CoV-2 infection but were significantly up-regulated upon IAV (fig. S11, I to K). Overall, our results highlight that under conditions in which SARS-CoV-2 infects AMs, it does so with comparatively low induction of IFN responses. Last, IFN-α treatment of AMs at 24 hours after infection did not significantly affect viral gene expression (Fig. 5L), suggesting that SARS-CoV-2 antagonizes IFN responses in AMs.

## DISCUSSION

The early host-pathogen response in SARS-CoV-2 infections has remained difficult to study, and thus, model systems have dominated our understanding. Here, we have used human lung slices, BAL samples from human lungs, and ETAs from individuals with COVID-19 ARDS to elucidate early pathogenesis and found a distinct tropism for myeloid populations, specifically AMs. AMs are sentinel immune cells in the distal lung relied on to robustly respond to environmental challenges, including pathogenic viruses. As we have shown with IAV, AMs reduce viral titers and sound the alarm that invasion is underway through the production of high levels of IFNs. This is distinctly the opposite with SARS-CoV-2 where AMs are directly targeted for productive infection via spike-ACE2 interactions. Given the scarcity of epithelial ACE2 in the distal lung, resident myeloid cells may be critical to viral expansion in the early phases of COVID-19.

It was only through the direct study of human lungs, including a complete representation of resident stromal and immune cell populations, that this discovery was possible. Human lung slices have been used by others to characterize SARS-CoV-2 tropism (*21*), including tropism (but not viral replication) in macrophages (*22*). Our approach to study the early responses to SARS-CoV-2 focused on lung resident cells, as immune cell recruitment from the vascular compartment is not modeled in the PCLS system. Also, PCLSs do not recapitulate SARS-CoV-2 infection by aerosol, instead allowing viral access to cells beyond the epithelial barrier. However, our focus on AMs (alveolar positioned sentinel cells) makes this less of a concern. Studies have established that in the evolution of COVID-19, monocytes are targeted through antibody-dependent mechanisms producing activation of the inflammasome and subsequent immunopathology (*7*, *23*). Our results differ in that we directly studied AMs, which are tissue-resident immune cells with a distinct ontogeny (*8*), and we studied the very early phase of infection when antibody-dependent mechanisms are not relevant. Viral entry in AMs is independent of antibody receptors or phagocytosis yet clearly dependent on ACE2-spike engagement.

While other studies have also detected SARS-CoV-2 in human AMs that could indicate potential for replication (*14*), we have directly tested for viral production by isolating human AMs and measuring viral release in the supernatant using the gold-standard plaque assay for quantifying concentrations of replication-competent lytic virions—yielding results that definitely point to viral expansion by AMs in this setting. We would also caution inferring results on viral replication from previous studies that used human monocyte-derived macrophages (*24*) or THP-1 cell lines engineered to express ACE2 (*25*), as these cells are very much different than primary human AMs and have different immune responses.

With respect to why this depot has been missed in the course of the pandemic, we note that our data and many other studies (see table S3) have not detected *ACE2* by scRNA-seq likely because of low transcript abundance and the sensitivity limitations of the technique (*16*). scRNA-seq has rapidly become a favorite method for rapid and automated RNA analysis despite its well-known dropout rate. In our study, using the more sensitive RT-qPCR method and flow cytometry staining with appropriate controls, ACE2 is clearly present in human AMs and functionally regulates SARS-CoV-2 entry. The variation in surface levels observed by flow cytometry may also point to the possibility that cells might modulate ACE2 from their surface in physiological settings. Future studies are needed to determine whether heterogeneity in ACE2 expression, perhaps driven by environmental influences or comorbidities, could be a disease-enabling pinch point in driving the severity of COVID-19 disease responses. Assessment of endotracheal samples from COVID-19 ARDS further validates that AMs harbor virus in patients, although it remains ethically impossible to directly measure that upon initial exposure.

Newly produced viruses can then contribute to SARS-CoV-2 dissemination and pathogenesis. This infection route also creates a myeloid depot, illustrated by the persistence of this infected AM population long after disease onset in the human COVID-19 ARDS cohort. Our study also elaborates a clear difference in immune responses for SARS-CoV-2 in AMs as compared to an exemplar influenza virus. While IAV was particularly potent at inducing IFNs and suppressing viral replication, SARS-CoV-2 induces opposite responses in AMs likely through transcriptional suppression of host IFN responses by the SARS-CoV-2 genome (*26*, *27*). A caveat is that we used a mouse-adapted influenza strain, and future studies should incorporate clinically important seasonal/pandemic influenza strains.

A key finding here is that AMs that are normally among the first line of defense are blunted for their responses, helping SARS-CoV-2 evade the immune system. Noting that these cells are migratory in the alveolar spaces (*28*), the silent but persistent infection of AMs could potentially lead to catastrophic viral spreading in the lung. Studies of others have previously also highlighted the finding of infected AMs in autopsy samples from deceased cases with COVID-19, supporting the generalized hypothesis that AMs could play a role in later phases of the disease (*29*), but our study differs in studying this in the first phase of lung infection. We also note that immune tropism is reminiscent of diseases such as HIV and in similar manner could present unique challenges for the elaboration of sterilizing immunity, particularly in cases with vey latent infections of these myeloid populations. Our findings are therefore critical as we consider potential long-lasting effects of this macrophage depot, which could also be linked to immune deviation, nonresolving critical illness, and long-term complications of COVID-19.

## MATERIALS AND METHODS

### Precision-cut lung slices

Human donor lungs were obtained from Donor Network West. Lung lobes were inflated using 2% low–melting point agarose and incubated at 4°C (*9*, *10*). After agarose consolidation, 1-cm^3^ lung tissue was placed on the precision compresstome VF-200 (Precisionary Instruments Inc.) for slicing. Slices (300 μm) were obtained and cultured in Dulbecco’s modified Eagle’s medium (DMEM) (UCSF Media Production), 1% penicillin/streptomycin (UCSF Media Production), and 10% fetal bovine serum (FBS) (Corning) in a 24-well plate. We focused on the distal alveolar lung for sampling.

### SARS-CoV-2 infections

Vero-TMPRSS2 cells (gift from M. Ott) were cultured in DMEM supplemented with 10% FBS, penicillin/streptomycin, and l-glutamine (Corning) in a humidified incubator at 37°C and 5% CO_2_. SARS-CoV-2 virus (USA-WA1/2020 strain) was provided by M. Ott and propagated in Vero-TMPRSS2 cells. SARS-CoV-2 B.1.617.2 (delta) variant was acquired from the California Department of Public Health, cultured in Vero-TMPRSS2 cells. For propagation, Vero-TMPRSS2 cells were infected with the SARS-CoV-2 virus, incubated at 37°C, 5% CO_2_, and at 72 hours, the supernatant was collected. The virus was aliquoted and stored at −80°C. All work was done under Biosafety Level 3 (BSL-3) conditions. Viral titer was quantified using a plaque assay in Vero cells (*30*). Briefly, 10-fold dilutions of the virus stock were added to Vero cells in a 12-well plate for 1 hour, after which an overlay of 1.2% Avicel RC-581 in DMEM was added. The cells were incubated at 37°C, 5% CO_2_ for 96 hours. The cells were fixed with 10% formalin, stained with crystal violet, and washed with water. The plaques were counted to determine the titer of the virus stock. For PCLS infection experiments, MOI was based on the average cell counts obtained after digestion of the slices.

### IAV infections

As a virus of reference, we used influenza A/Puerto Rico/8/34 (*PR8*, H1N1) virus labeled with Venus to infect lung slices and BAL cells. PR8-Venus IAV was a gift from Y. Kawaoka (University of Wisconsin-Madison). The virus was produced in pathogen-free fertilized chicken eggs (Charles River) as published (*31*). In brief, eggs were kept in an egg turner for 10 days. PR8-Venus IAV was injected into the allantoic cavity. Infected chicken embryos were incubated with the virus for 48 hours. Allantoic fluid was harvested, filtered, and snap-frozen in liquid nitrogen. Titers were determined with a hemagglutination assay. The work was done under BSL-2 conditions.

### Immunofluorescence imaging

After infection, PCLS were fixed in 4% PFA for at least 30 min. After saturation in PBS with 1% bovine serum albumin (BSA), slices were stained with anti-CD45 (HI30, 304056, BioLegend), anti-spike (40150-R007, Sino Biological), anti-ACE2 (bs-1004R, BIOSS) anti-dsRNA (J2, 10010, Scicons), or anti–EpCAM-AF488 (9C4, 324210, BioLegend) for 1 hour in PBS with 1% BSA media at room temperature. PCLS were washed in PBS, counterstained with 4′,6-diamidino-2-phenylindole, and attached to plastic coverslips using Vetbond (3M). Confocal imaging was performed using a Nikon A1R laser scanning confocal microscope with NIS-Elements software and a 16X Long-Working Distance (LWD) water dipping objective. Images were taken at more than 25 μm deep inside the slices to avoid the cutting artifact. Images (50 to 100 μm thick) with a z-step of 1.5 μm were taken and analyzed using Imaris (Bitplane).

### Flow cytometry

At selected time points, PCLS were dissociated using deoxyribonuclease (DNase) (4 μg/ml) and collagenase IV (200 U/ml) at 37°C for 30 min. Cells were filtered, centrifuged (300*g*, 5 min), and stained for viability and surface markers (table S4). After fixation and permeabilization (BD Cytofix/Cytoperm), cells were stained for spike and dsRNA (table S5). Data were collected using the BD LSRII Cytometer and analyzed using FlowJo version 10 (BD Biosciences).

### Bronchoalveolar lavage

A BAL was done in a human lung lobe using ice-cold PBS. BAL cells were filtered, and centrifuged (300*g*, 5 min), and red blood cells were lysed. BAL cells were centrifuged (300*g*, 5 min) and plated in 24-well plates at 5 ×10^5^ cells per well in DMEM, 1% PS, 10% FBS containing rhM-CSF and rhGM-CSF (50 ng/ml; Peprotech). For mechanism of entry experiments, BAL cells were treated for 2 hours before infection. ACE2 blocking antibody (AF933, R&D Systems), RBD blocking antibody (MAB105802, R&D Systems), and CD16 blocking antibody (555403, BD Biosciences) were used at 10 μg/ml. Cytochalasin D (Sigma-Aldrich) was used at 1 μM. SARS-CoV-2 was added to the cells at MOI 0.1 or 1. After 48 hours of infection, cells were recovered and stained for viability and surface markers (table S5). After fixation and permeabilization, cells were stained for intracellular spike expression. In selected experiments, AMs (live, EpCAM-, CD3-, CD19-, CD45^+^, HLA-DR^+^, CD169^+^) were flow-sorted before infection with SARS-CoV-2 using FACSAria Fusion (BD Biosciences). As AM are auto fluorescent, flow cytometry stainings were compared to either unstained or untreated cells.

In selected experiments, “plated AMs” were isolated from BAL. Briefly, BAL cells were plated at 0.5 × 10^6^ cells/cm^2^ in presence of FBS and rhGM-CSF and rhM-CSF (50 ng/ml for both). Cells were incubated for 1 hour at 37°C and 5% CO_2_. Nonadherent cells were removed by washing twice with warm media. AMs were recovered using PBS 5 mM EDTA and centrifuged (250*g*, 10 min). Last, cells were plated at 0.2 × 10^6^ cells/cm^2^. After a rest period of minimum 2 hours, cells were infected with either IAV or SARS-CoV-2.

### Virus replication assay

BAL cells were infected for 48 hours either with SARS-CoV-2 or IAV-Venus at MOI 0.1 or 1. At the end of the incubation, cell-free supernatant was recovered. This solution was used as inoculum for Vero-TMPRSS2 cells (SARS-CoV-2) or MDCK cells (IAV-Venus). After 24 hours of incubation, cells were recovered and infection was assessed by flow cytometry. Virus titers were also determined using plaque assay (described previously) with either Vero-TMPRSS2 cells (SARS-CoV-2) or MDCK cells (IAV-Venus).

### Western blot

Cells were lysed in RIPA lysis buffer (no. 89900, Thermo Fisher Scientific) containing protease and phosphatase inhibitors HALT (no. 78430, Thermo Fisher Scientific). The total protein concentration was measured by a bicinchoninic acid protein assay kit, according to the manufacturer’s instructions (no. 23225, Thermo Fisher Scientific). Protein samples (25 μg) were resolved using a mini 4 to 12% SDS–polyacrylamide gel electrophoresis gels (NP0322BOX, Thermo Fisher Scientific) and then transferred to nitrocellulose membranes (IB301002, Thermo Fisher Scientific). The membranes were blocked for 3 hours using TBST blocking buffer containing 0.1% Tween 20 with 2% BSA (TBST-BSA). Next, membranes were incubated overnight with primary antibodies at 1:1000 diluted in TBST-BSA: anti-ACE2 AC18F (no. 30582, Cayman Chemical), sn0754 (no. MA5-32307, Thermo Fisher Scientific), or glyceraldehyde-3-phosphate dehydrogenase. (GAPDH) diluted at 1:10,000 (no. 10494-1-AP, Proteintech). Membranes were washed three times with TBST and then were incubated in secondary antibody at 1:5000 diluted in TBST-BSA: anti-mouse (115-035-003, Jackson ImmunoResearch) or anti-rabbit (no. 31460, Invitrogen), respectively. Femto ECL reagent (no. 20-302, Geneseesci) was used to detect chemiluminescent signal on ImageQuant LAS4000 (GE HealthCare).

### RT-qPCR gene expression assay

RNA extraction was accomplished using the RNA extraction kit from Qiagen (RNeasy Micro kit) including a step of DNA digestion by DNase I. Single-strand cDNA was synthesized from up to 1 μg of total RNA from each sample with the SuperScript IV VILO (Invitrogen), including a DNA digest step using ezDNase. mRNA levels were determined by RT-qPCR with a ViiA 7 real-time PCR system (Applied Biosystems). Taqman primers were selected on the basis of best coverage: *ACE2*: Hs01085333_m1 and Hs00222343_m1, *GAPDH*: Hs02786624_g1, *ISG15*: Hs01921425-s1, *IRF7*: Hs01014809-g1, *IFI16*: Hs00986757-m1, *IFITM3*: Hs03057129-s1, *IFNG*: Hs00989291-m1, *IFNA*: HS03044218-g1, IAV: Vi99990011_po, SARS-CoV-2 N gene: Vi07918637_s1, SARS-CoV-2 S gene: Vi07918636_s1, Thermo Fisher Scientific). PCR was carried out by using 30 to 100 ng of reverse-transcribed total RNA with Taqman universal PCR Master Mix (Applied Biosystems) in a final volume of 10 μl. Each reaction was performed in triplicate in 384-well plates. The thermal protocol consisted of an initial denaturation step at 95°C followed by 45 cycles of denaturation at 95°C for 15 s and primer annealing and extension at 60°C for 60 s. Melting curves were generated for each amplified cDNA to check the specificity of the reactions. mRNA concentration was normalized to the level of GAPDH.

### Single-cell RNA sequencing

PCLS [human lung donors (see table S1), three technical replicates per condition] were dissociated as described above, and dead cells were removed using Miltenyi Dead Cell Removal Kit (130-090-101, Miltenyi Biotec). For the multiplexing purpose, cells were then labeled with lipid-modified oligonucleotides (LMO) and barcode oligos using the Multi-seq technique (*32*). Cells were counted and the targeted cell number for loading was 8000 cells per sample. Encapsulation (10X) and library construction were done using Chromium Next GEM Single Cell 3’ Reagent Kits v3.1 per the manufacturer’s instruction. Multi-seq library preparation was done as previously described (*32*). Libraries were mixed at an approximate 10:1 molar ratio of gene expression to LMO barcodes for sequencing. The sequencing was done on the Illumina NovaSeq 6000 using 10X Genomics recommended sequencing parameters.

### Data preprocessing of 10x Genomics Chromium scRNA-seq data

Sequencer-generated bcl data (Gene expression and Lipid Hashtag) was demultiplexed into individual fastq libraries using the mkfastq command on the Cellranger 3.0.2 suite of tools (https://support.10xgenomics.com). Feature-barcode matrices for all samples were generated using the Cellranger count command. Briefly, raw gene-expression fastqs were aligned to a custom cellranger reference containing the human GRCh38 reference genome annotated with Ensembl v85, the IAV genome corresponding to the strain used in the infections (RefSeq assembly GCF_000865725.1), and the SARS-CoV-2 genome (NCBI reference NC_045512.2). Lipid Hashtag fastqs were processed to count the incidences of each expected index per cell. Feature-barcode matrices were read into Seurat 4.0.1 (*33*) and poorly captured genes (in <3 cells) were dropped from the analyses. Matrices were further filtered to remove events with greater than 20% mitochondrial content, events with greater than 50% ribosomal content, or events with fewer than 100 total genes.

### Data quality control and normalization

The gene expression count matrices were normalized, and variance stabilized using negative binomial regression using the scTransform algorithm (*34*) in the Seurat package. Cellular mitochondrial content, ribosomal content, and cell cycle state were regressed out of the data at this stage to prevent any confounding signal. The normalized matrices were transformed into a lower subspace using Principal components analysis (PCA) and 50 PCs per samples were used to generate Uniform Manifold Approximation and Projection (UMAP) visualizations. Cell clustering via expression was conducted using the Louvain algorithm. Cluster identities were assigned using Gene scores generated via the Seurat AddModuleScore function on a list of gene sets obtained from the Human Lung Cell Atlas.

### Demultiplexing of pooled single-cell libraries

In Lipid hashtagged libraries, the raw lipid tag counts were normalized using the Centered Log Ratio method where HTO counts are divided by the geometric mean for that HTO across all cells and then log normalized. The resulting matrix was demultiplexes into donor samples using the Seurat HTODemux function (*34*) using default parameters.

### Data integration and batch correction and availability

scTransformed count matrices from all samples were integrated together using the in-built integration method provided by Seurat. The following commands were run in order, to integrate datasets “SelectIntegrationFeatures” to identify shared “anchor” features for integration, “PrepSCTIntegration” to subset objects based on identified anchor features, “FindIntegrationAnchors” to identify anchor points between datasets on the basis of the anchor features, and last, “IntegrateData” to integrate the datasets based on the computed anchors. Downstream processing (PCA, UMAP, clustering and cluster assignment) was conducted similarly to the individual libraries.

### ETA samples

ETA samples were prospectively collected from seven adults requiring mechanical ventilation for the ARDS from COVID-19 as part of the COVID Multiphenotyping for Effective Therapies (COMET) study, as previously described (*35*). Human participants were enrolled at two tertiary care hospitals in San Francisco, CA (UCSF Medical Center, Zuckerberg San Francisco General Hospital). ETA samples were collected within 1 to 40 days after intubation for scRNA-seq. Please see (*35*) for data availability for these samples. See table S2 for additional details.

### Statistics

Statistical analysis was performed using GraphPad Prism v7.0e. For PCLS experiments (Fig. 1), significance was assessed using analysis of variance (ANOVA) with Sidak’s multiple comparison test. For BAL cells and AM experiments, each dot represents the mean of two to three replicates from a single donor. Donors are color-coded across experiments. Paired multiple group comparisons were analyzed by one-way ANOVA with Dunnett’s multiple comparisons. Ratio paired *t* test was used to compare two paired groups (ACE2 blockade, virus propagation assay, plaque assay).

### Study approval

Experiments using human samples were approved under research protocol no. 20-30497 by the University of California, San Francisco Institutional Review Board.

## Supplementary Materials

### This PDF file includes:

The UCSF COMET Consortium Figs. S1 to S11 Tables S1 to S5 References
