# Supplementary material for: Immediate myeloid depot for SARS-CoV-2 in the human lung
Source: Sci Adv. 2024 Jul 31;10(31):eadm8836. doi: 10.1126/sciadv.adm8836 (PMC11290487; doi:10.1126/sciadv.adm8836)
Supplement: Supplementary file 1 — The UCSF COMET Consortium Figs. S1 to S11 Tables S1 to S5 References [file sciadv.adm8836_sm.pdf]

Science Advances
AAAS

# Supplementary Materials for

## Immediate myeloid depot for SARS-CoV-2 in the human lung

Mélia Magnen *et al.*

Corresponding author: Matthew F. Krummel, matthew.krummel@ucsf.edu;
Mark R. Looney, mark.looney@ucsf.edu

*Sci. Adv.* **10**, eadm8836 (2024)
DOI: 10.1126/sciadv.adm8836

**This PDF file includes:**

The UCSF COMET Consortium
Figs. S1 to S11
Tables S1 to S5
References

**Collaborators**

**The UCSF COMET Consortium**

Cai Cathy, Bushra Samad, Suzanna Chak, Rajani Ghale, Jeremy Giberson, Ana Gonzalez, Alejandra Jauregui, Deanna Lee, Viet Nguyen, Kimberly Yee, Yumiko Abe-Jones, Logan Pierce, Priya Prasad, Pratik Sinha, Alexander Beagle, Tasha Lea, Armond Esmalii, Austin Sigman, Gabriel M. Ortiz, Kattie Raffel, Chayse Jones, Kathleen Liu, Walter Eckalbar, Billy Huang, Norman Jones, Jeffrey Milush, Ashley Byrne, Saherai Caldera, Catherine DeVoe, Paula Hayakawa Serpa, Eran Mick, Mayra Phelps, Alexandra Tsitsiklis, K. Mark Ansel, Stephanie Christenson, Gabriela K. Fragiadakis, Andrew Willmore, Sidney A. Carrillo, Alyssa Ward, Kirsten N. Kangelaris, Simon J. Cleary, Zoe M. Lyon, Vincent Chan, Nayvin Chew, Alexis Combes, Tristan Coureau, Kamir Hiam, Kenneth Hu, Billy Huang, Nitasha Kumar, Divya Kushnoor, David Lee, Yale Liu, Salman Mahboob, Priscila Munoz-Sandoval, Randy Parada, Gabriella Reeder, Alan Shen, Yang Sun, Sara Sunshine, Jessica Tsui, Juliane Winkler, Peter Yan, Michelle Yu, Shoshana Zha, Didi Zhu.

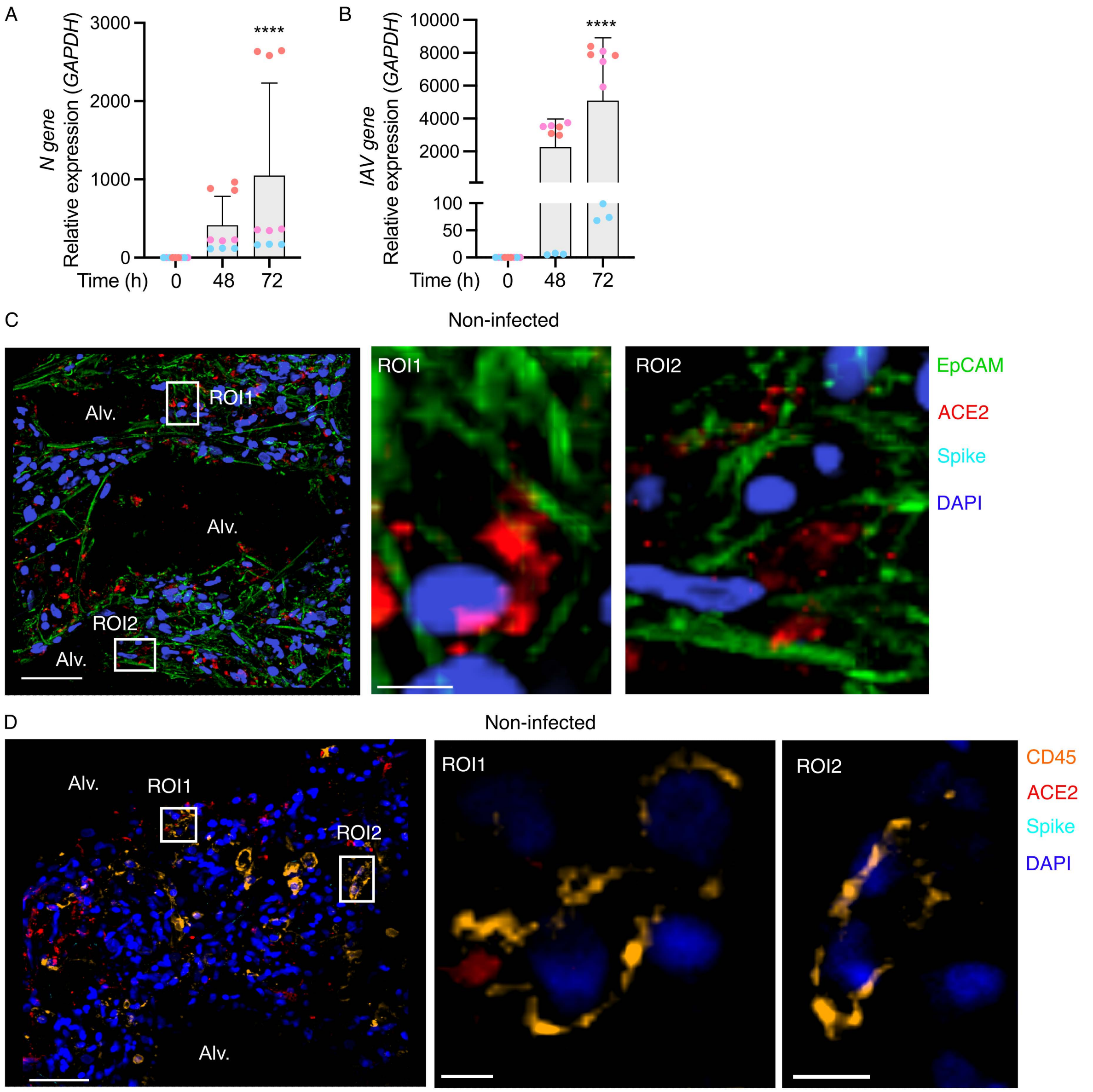


**Figure S1. Virus replication in infected PCLS.** (A-B) PCLS were infected with SARS-CoV-2 (A) or IAV (B) and recovered at 48 and 72h post-infection. Viral replication was assessed by RT-qPCR. Individual donors color-coded and Mean +/- SD. ****$p<0.0001$. Confocal images were obtained from non-infected controls (scale bar = 50 µm). Zoomed area is marked by the white rectangles. For each zoomed area, slices appear on the side (scale bar = 5 µm in (C) and 10 µm in (D). (C) PCLS stained for DAPI (dark blue), EpCAM (green), ACE2 (red) and spike (light blue). (D) PCLS stained for DAPI (dark blue), CD45 (orange), ACE2 (red) and spike (light blue).

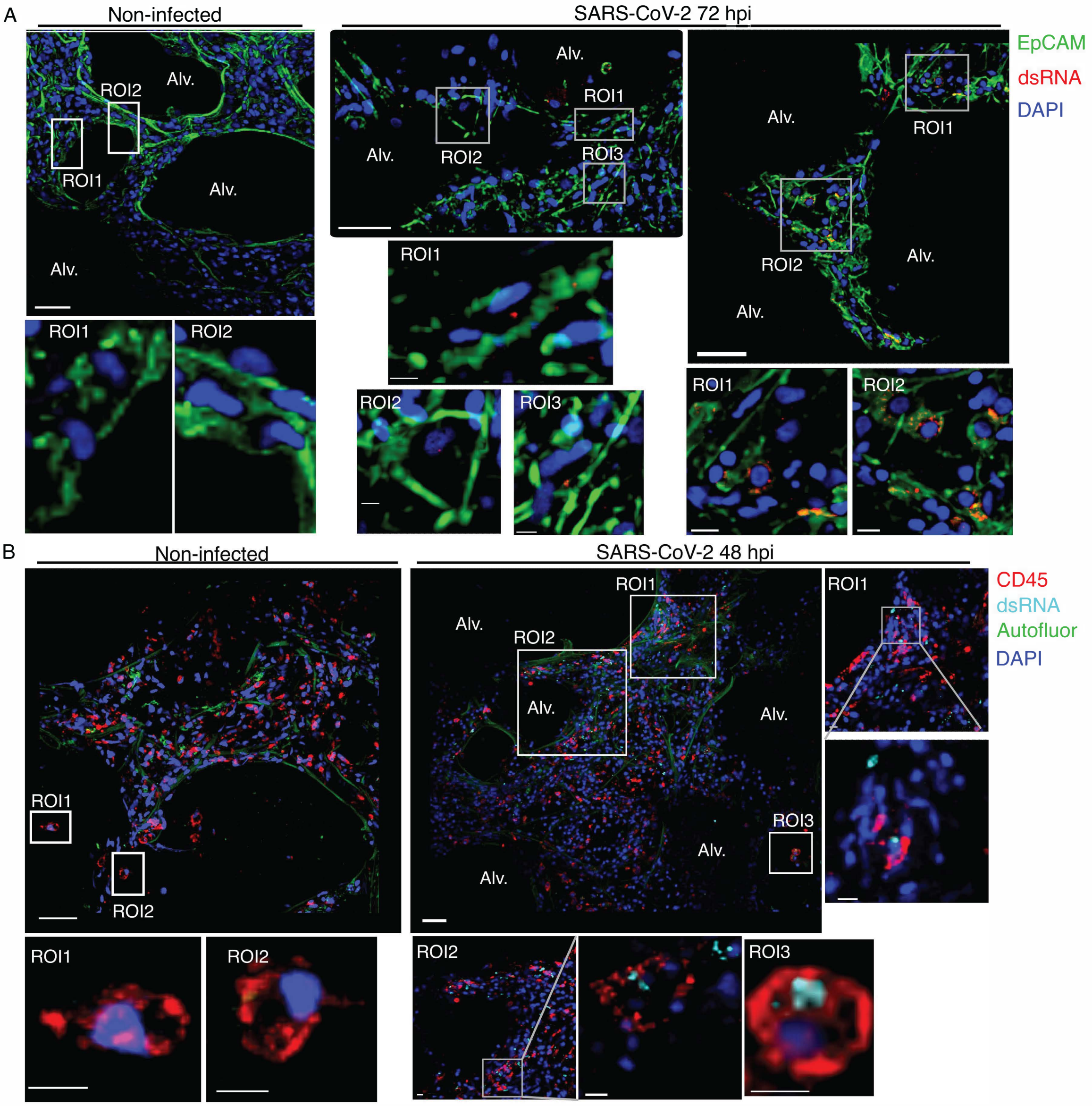


**Figure S2. SARS-CoV-2 infection drives dsRNA production in both epithelial and immune cells in PCLS.** PCLS were infected with SARS-CoV-2 and used for confocal imaging. Alveolar spaces (Alv.) are indicated in the large image (scale bar = 50 µm). Zoomed area is marked by the white rectangle. For each zoomed area, z-stacks appear on the side (scale bar = 10 µm). (A) PCLS stained for DAPI (dark blue), EpCAM (green) and dsRNA (red). (B) PCLS stained for DAPI (dark blue), CD45 (red) and dsRNA (light blue). Autofluorescence appears in green.

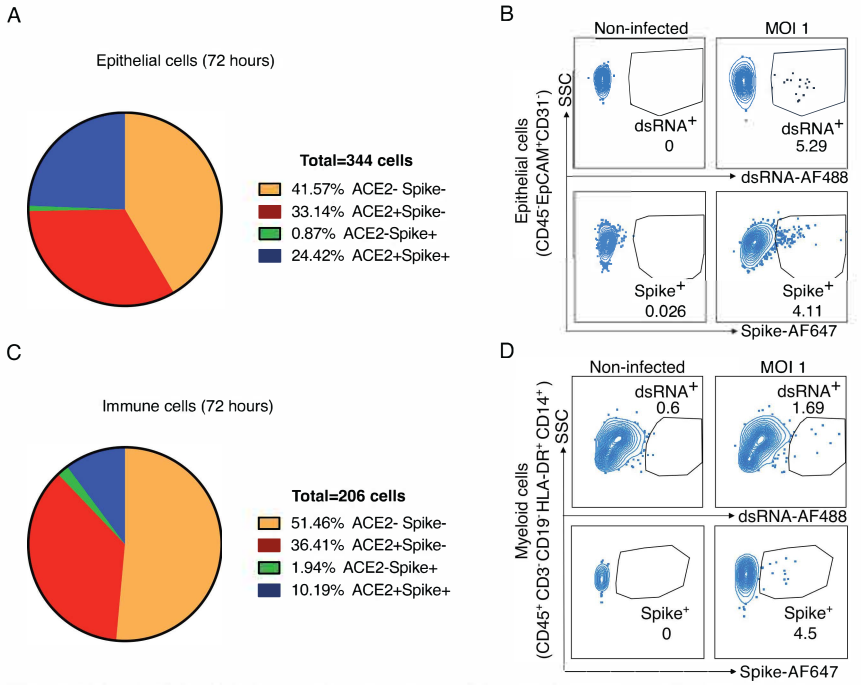


**Figure S3. SARS-CoV-2 infection of epithelial and immune cells occurs mainly in ACE2+ cells.** SARS-CoV-2-infected PCLS were used for confocal imaging. EpCAM+ (A) or CD45+ (C) cells were analyzed for ACE2 and spike staining. (A, C) Counts are presented in pie charts. Data are pooled from three donors. (B, D) SARS-CoV-2-infected PCLS were dissociated and stained for intracellular dsRNA and spike. Epithelial (B) and myeloid (D) cell infection was assessed by flow cytometry.

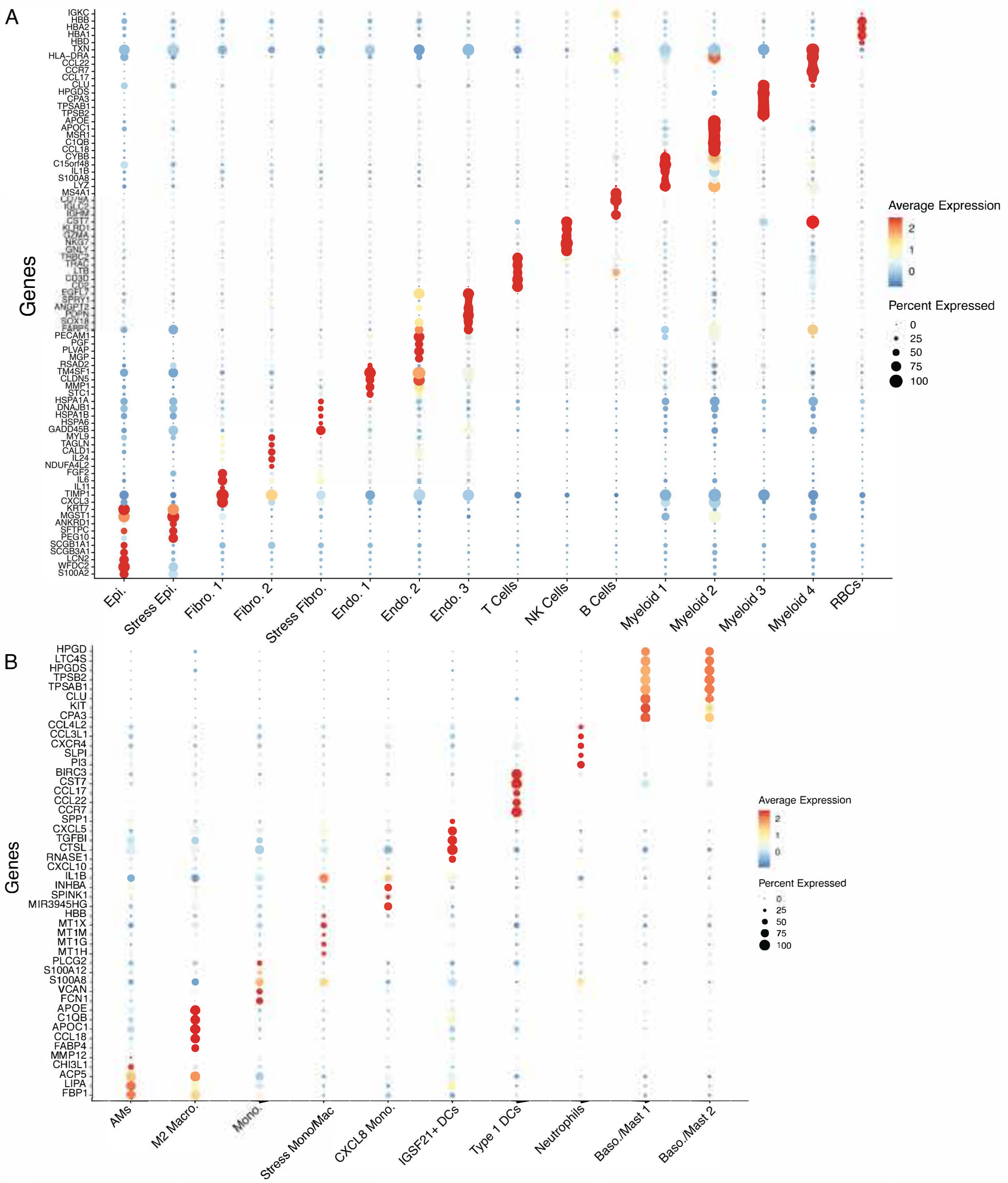


**Figure S4. Cell type annotations for scRNA-seq in human PCLSs.** (A) A dot plot describing the expression of the top 5 genes per identified cluster in the human PCLS dataset, used to assign cell type annotations. (B) A dot plot describing the expression of the top 5 genes per identified myeloid subcluster in the human PCLS dataset, used for myeloid cell type annotations.

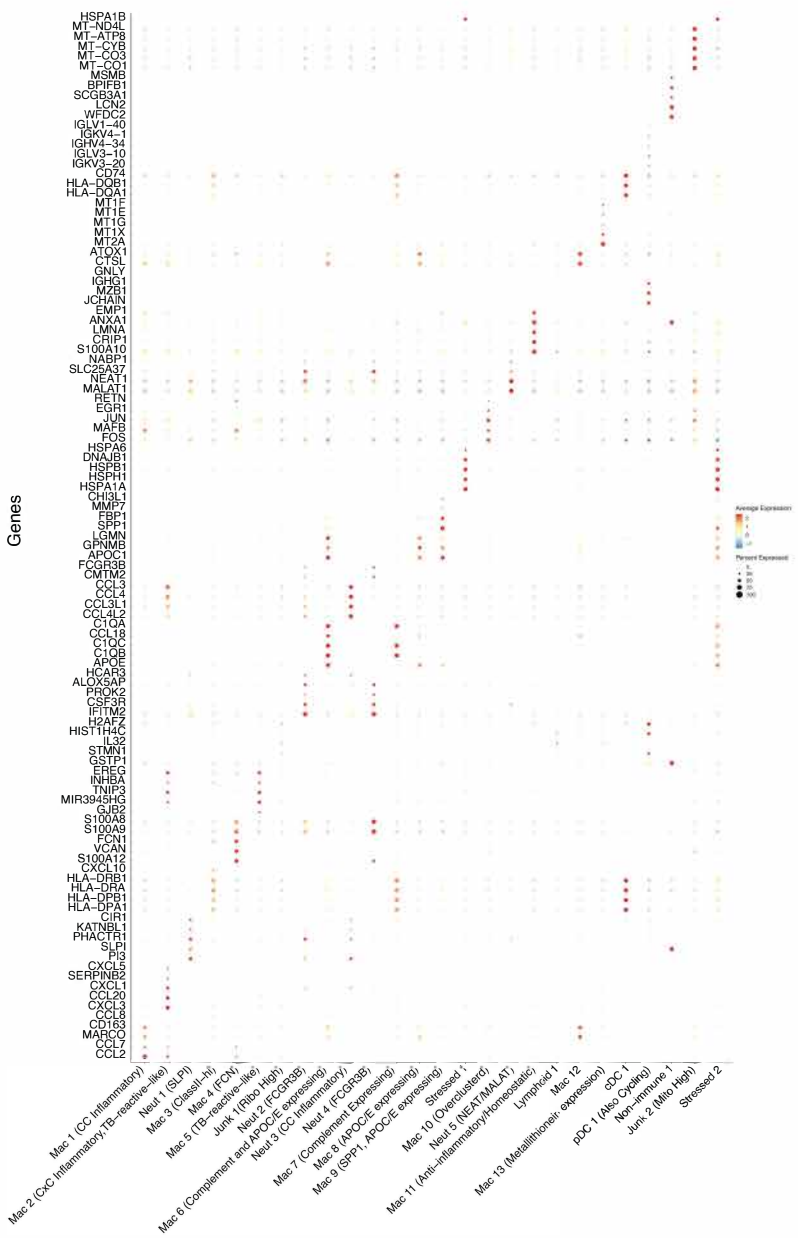

**Figure S5. Cell type annotations for scRNA-seq in human ETA samples.** A dot plot describing the expression of the top 5 genes per identified cluster in the human ETA dataset, used to assign cell type annotations.

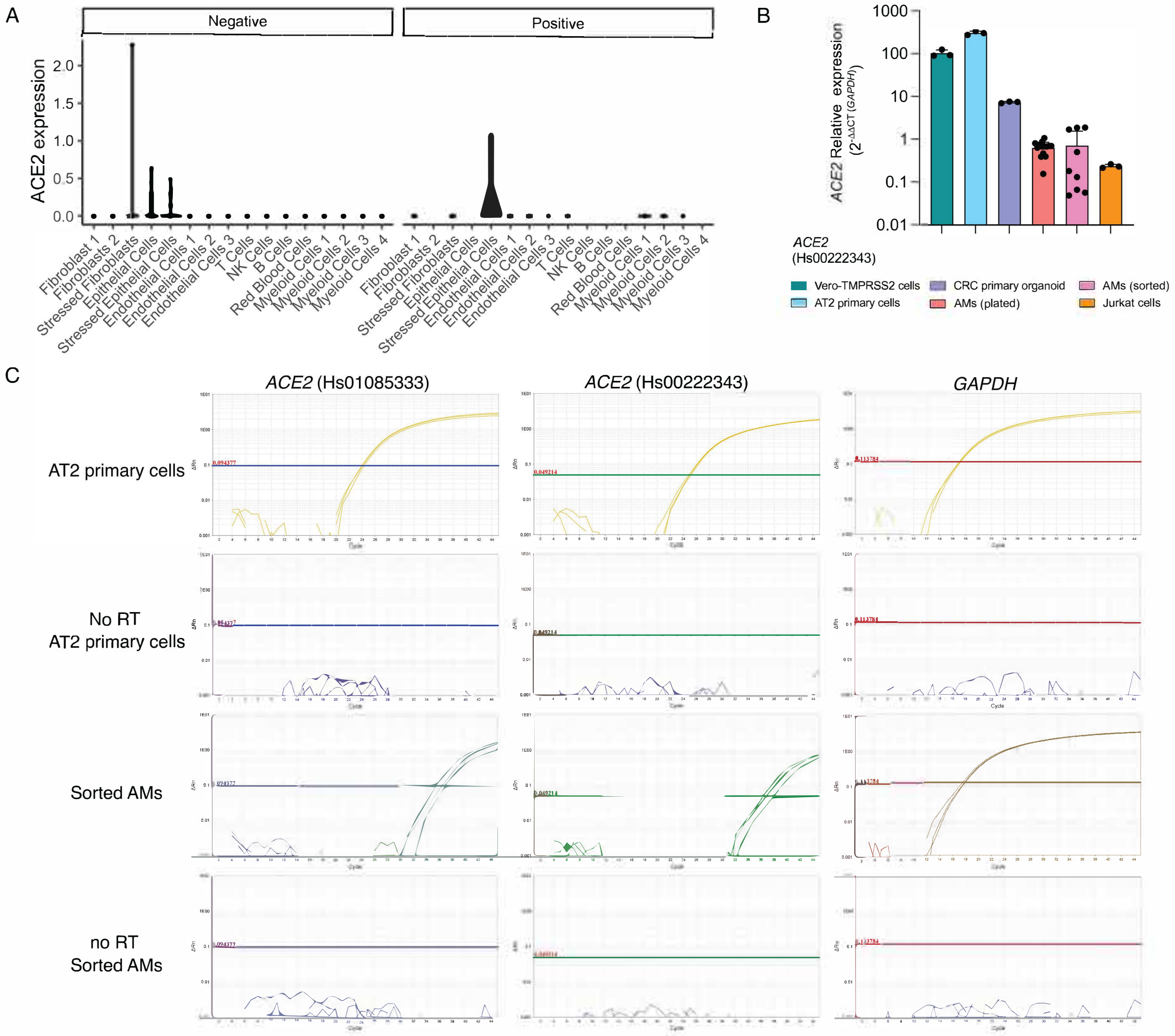


**Figure S6. ACE2 transcript is expressed in human alveolar macrophages.** A) *ACE2* expression in PCLS scRNA-seq. Cells are grouped according to their SARS-CoV-2 status (positive or negative). (B) *ACE2* transcript expression was assessed by RT-qPCR in Vero-TMPRSS2 cells (green), AT2 primary cells (blue), colorectal cancer (CRC) organoid (violet), Jurkat cells (orange) and AMs from human BAL (red (plated) and pink (flow-sorted), n= 3 donors). Relative mRNA expression is displayed as $2^{-\Delta\Delta CT}$ relative to GAPDH for probe Hs00222343. Vero-TMPRSS2 are set at 100. Mean +/- SD. (C) Amplification curves for AT2 primary cells and sorted AMs with their corresponding negative control (no RT).

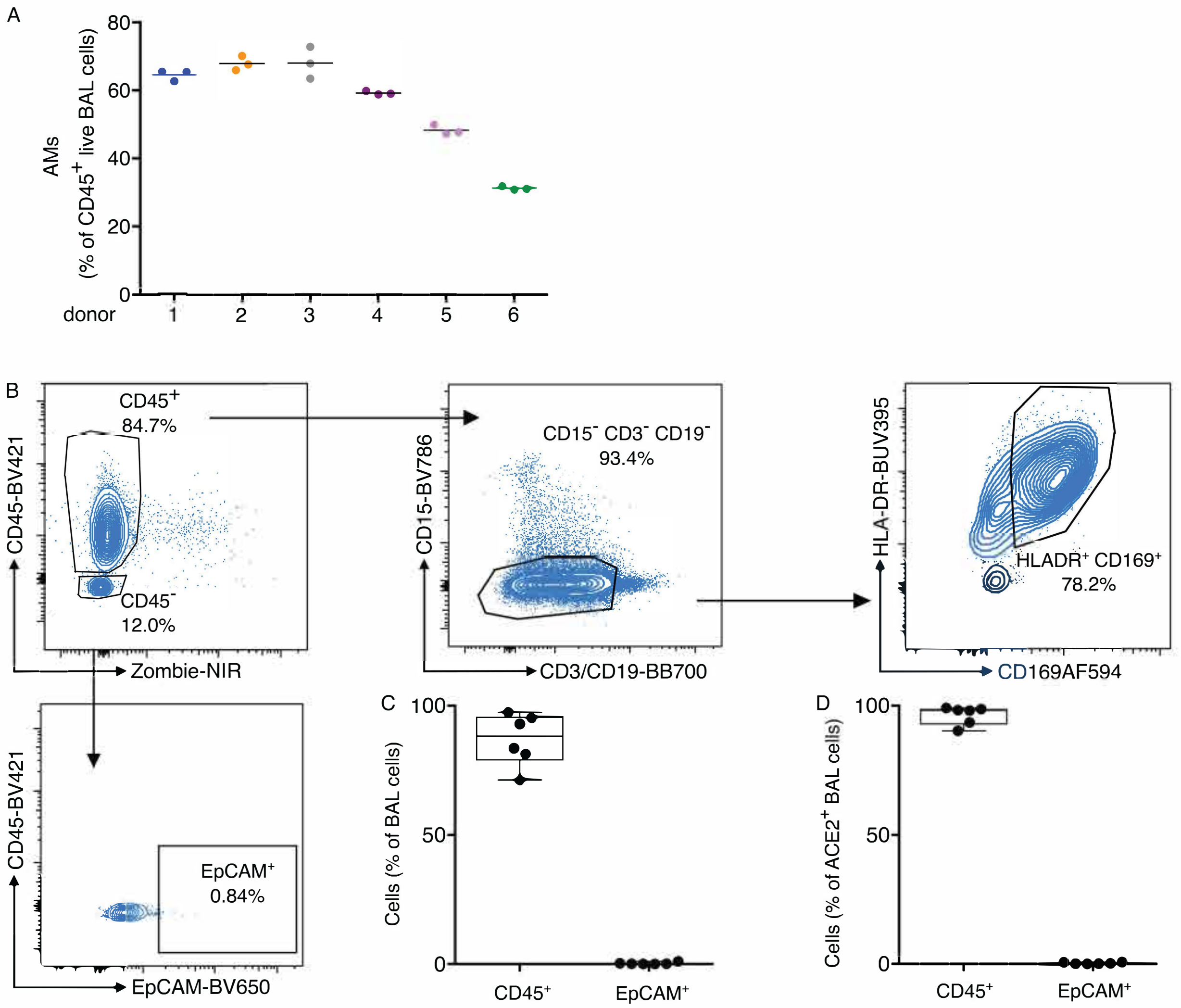


**Figure S7. Human BAL cell profile.** (A) Proportion of AMs (CD45+ CD15-, CD3- CD19- HLA-DR+ CD169+) amongst total, live BAL cells (n=6 donors). (B) Flow cytometry gating of human BAL cells. (C, D) Proportions of leukocytes (CD45+) and epithelial cells (CD45- EpCAM+) in total and ACE2+ BAL cells (n=6 donors).

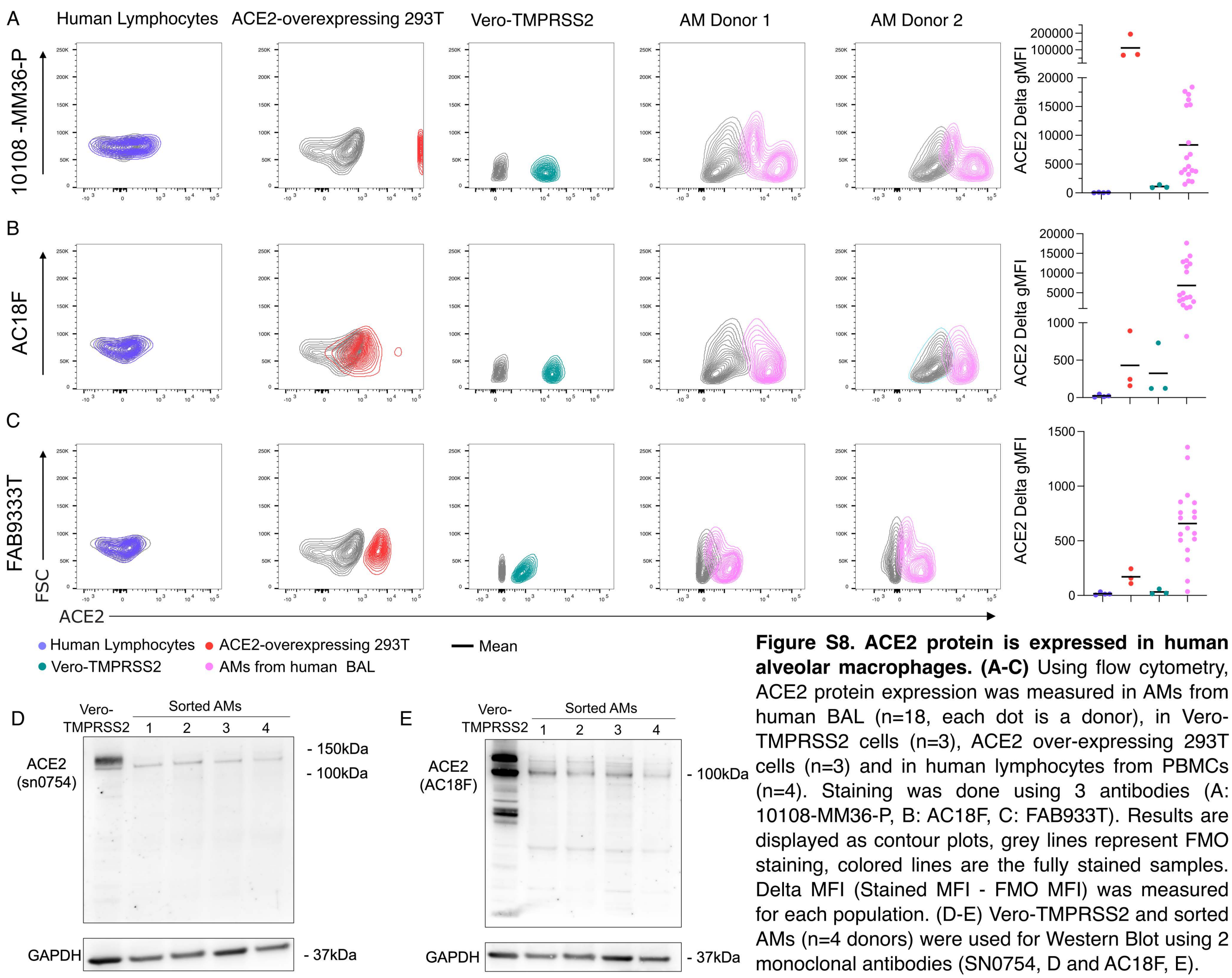


**Figure S8. ACE2 protein is expressed in human alveolar macrophages. (A-C)** Using flow cytometry, ACE2 protein expression was measured in AMs from human BAL (n=18, each dot is a donor), in Vero-TMPRSS2 cells (n=3), ACE2 over-expressing 293T cells (n=3) and in human lymphocytes from PBMCs (n=4). Staining was done using 3 antibodies (A: 10108-MM36-P, B: AC18F, C: FAB933T). Results are displayed as contour plots, grey lines represent FMO staining, colored lines are the fully stained samples. Delta MFI (Stained MFI - FMO MFI) was measured for each population. (D-E) Vero-TMPRSS2 and sorted AMs (n=4 donors) were used for Western Blot using 2 monoclonal antibodies (SN0754, D and AC18F, E).

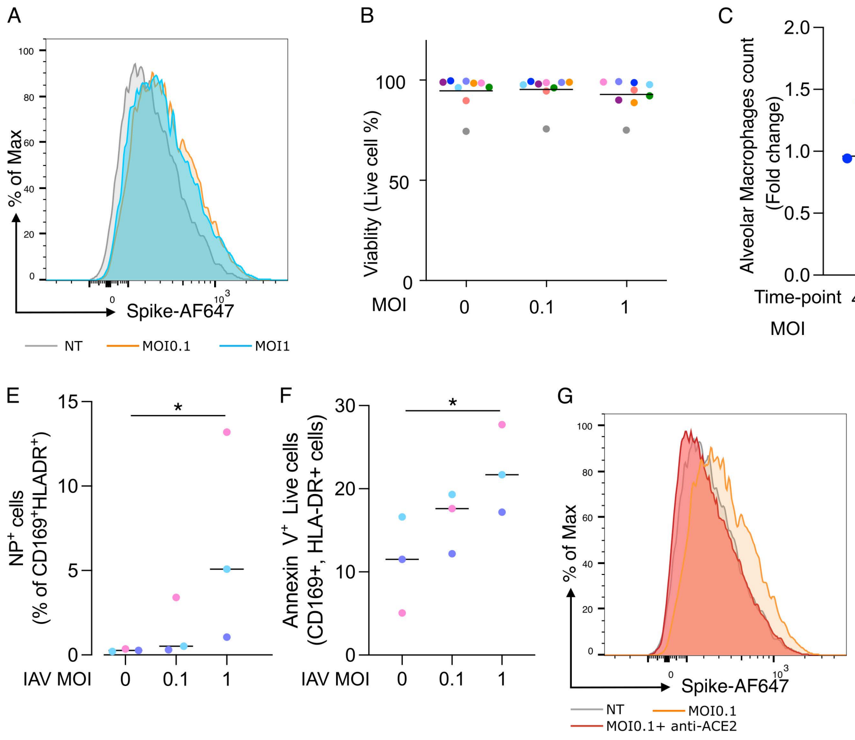


**Figure S9. SARS-CoV-2 infection in human BAL cells.** (A) Representative intracellular spike flow cytometry staining on AM population after 48h infection by SARS-CoV-2 at indicated MOI. (B) Viability of BAL cells infected for 48h with SARS-CoV-2 at MOI 0.1 or 1 or control (n=9 donors). (C) #AMs are expressed in fold change from uninfected control after 48 and 96h infection with SARS-CoV-2 at MOI 0.1 or 1 (n=3 donors). (D) Apoptosis was assessed using Annexin V staining and flow cytometry analysis (n= 4 donors). (E-F) BAL cells were infected with IAV at MOI 0.1 or 1 for 48 h. Cells were stained for viral nucleoprotein (NP, E) and Annexin V (F) (n= 3 donors). (G) Representative intracellular spike flow cytometry staining on AM population after 48h infection by SARS-CoV-2 at MOI 0.1, +/- ACE2-blocking antibody (AF933). * $p<0.05$.

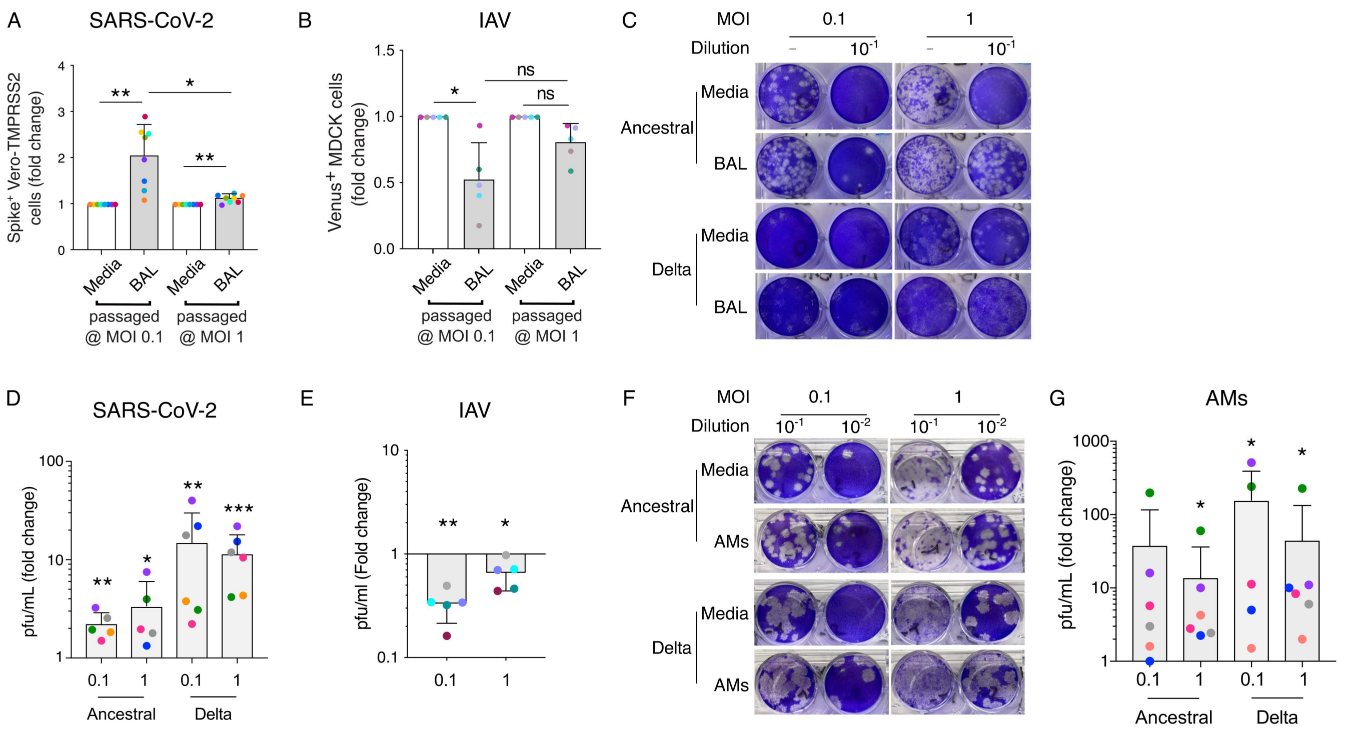


**Figure S10. Infection of BAL cells and flow-sorted AMs with SARS-CoV-2 variants.** (A) BAL cells were infected with SARS-CoV-2 for 48h at MOI 0.1 and 1 (BAL). As a control, SARS-CoV-2 was incubated in cell culture media alone (Media). Cell-free supernatant was used to infect Vero-TMPRSS2 cells. At 24h after infection, Vero-TMPRSS2 were stained for intracellular spike expression. Using flow cytometry, infection rate was measured (n=8 donors, mean ± SD). Results appear in fold change compared to media incubation. (B) Similarly, IAV-Venus was used to infect BAL cells or incubated with media. Cell-free supernatant was used to infect MDCK cells. At 24h, MDCK were recovered for flow cytometry (n=5 donors, mean ± SD). Venus expressing cells percentage was measured and appears as a fold change from media incubation. (C, D, E) Viral titer was determined by plaque assay. (D, E) Results appear as fold change from media incubated virus (n=5-6 donors, mean ± SD). (F, G) BAL AMs (live, EpCAM-CD45+CD3-CD19-HLA-DR+CD169+) were flow-sorted and SARS-CoV-2 variants were incubated with either AMs (MOI 0.1 - 1) or media for 48h. Cell free supernatant was used for plaque assay. (G) Results are expressed as fold-change compared to media incubation (n=5-6 donors, mean ± SD). ns not significant, *$p<0.05$, **$p<0.01$, ***$p<0.001$.

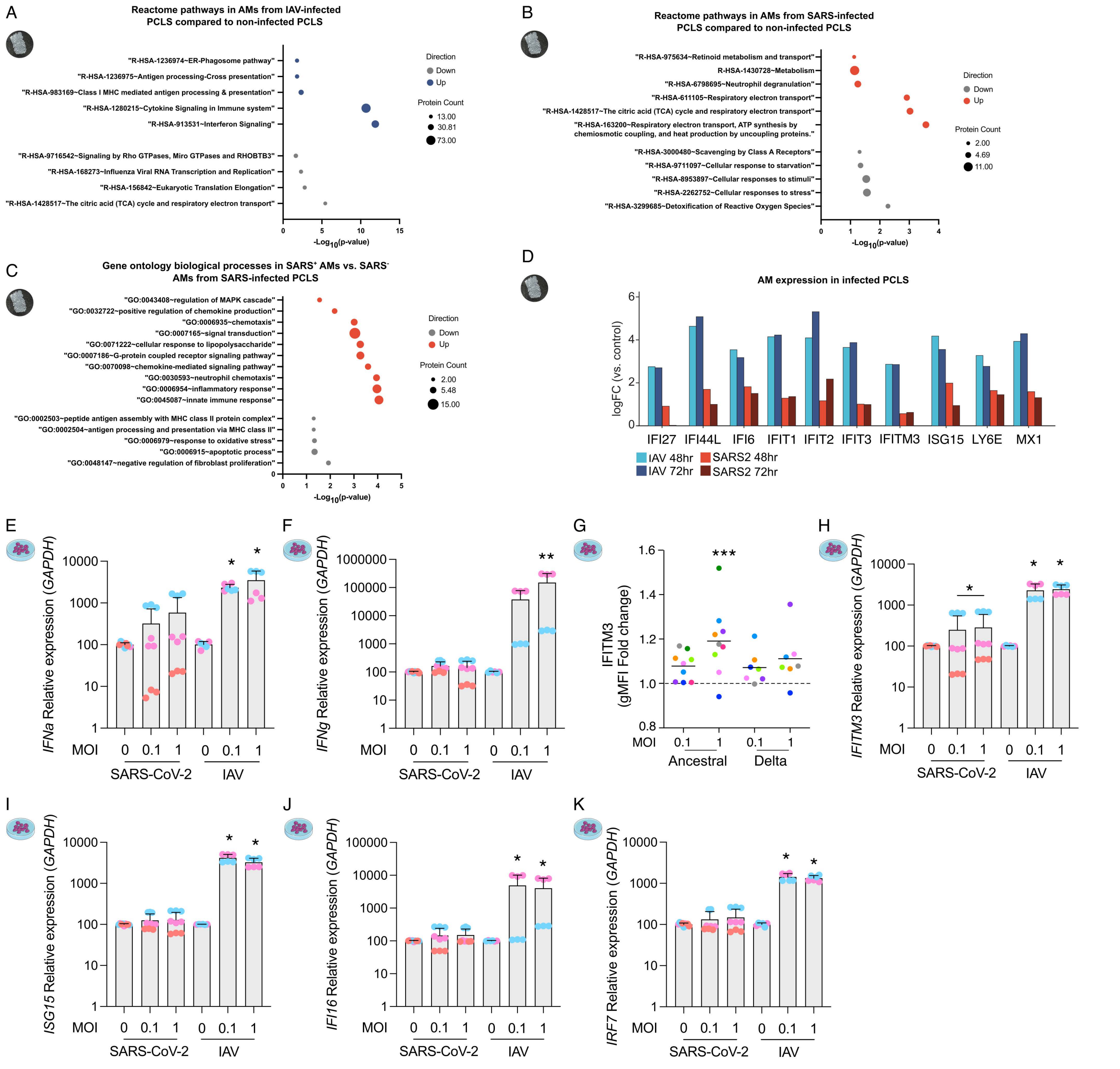


**Figure S11. SARS-CoV-2 induces blunted interferon responses in alveolar macrophages.** (A-C) PCLS AM pathway analysis at 48h post-infection. (A) AMs from IAV-infected PCLS compared to non-infected PCLS. (B) AMs from IAV-infected PCLS compared to non-infected PCLS. (C) Within SARS-CoV-2-infected PCLS, comparison of SARS-CoV-2 positive AMs compared to SARS-CoV-2 negative AMs. (D) Log2 fold change of select ISGs in the PCLS experiment in IAV- and SARS-CoV-2-infected AMs at 48 and 72h vs control. (E-F, H-K) Plated AMs were infected with SARS-CoV-2 or IAV at MOI 0.1 or 1 for 48h (n= 3 donors). (E) *IFNa* and (F) *IFNg* gene expressions were measured by RT-qPCR. (G) IFITM3 staining in AMs after infection with SARS-CoV-2 (Ancestral, delta) at MOI 0.1 - 1 (n=7-9 donors). Results appear in fold change compared to uninfected cells. (H) *IFITM3*, (I) *ISG15*, (J) *IFI16*, and (K) *IRF7* gene expression were measured in plated AMs at 48h post-infection. *p<0.05, **p<0.01, ***p<0.001.

## Table S1. Human lung donor demographics.

| Donor | Gender | Race/ Ethnicity | Age | Cause of death | Donation | Comorbidities | Smoking (>20PY) | Experiment Type |
|---|---|---|---|---|---|---|---|---|
| **1** | M | White | 63 | Anoxia | DBD | Hypertension, Pulmonary nodules | yes | BAL |
| **2** | F | White | 49 | Anoxia | DCD | Asthma, COPD, Sepsis pneumonia | yes | PCLS flow, PCLS imaging |
| **3** | F | White | 37 | Anoxia | DBD | None | no | PCLS flow, PCLS imaging |
| **4** | M | White | 51 | Stroke | DBD | Diabetes, CAD, Hypertension | no | PCLS flow, PCLS imaging |
| **5** | M | White | 47 | Stroke | DCD | None | no | PCLS flow, PCLS imaging |
| **6** | M | Asian/White | 32 | Head Trauma | DBD | Diabetes | no | PCLS flow |
| **7** | F | Hispanic | 44 | Stroke | DBD | None | no | PCLS flow, PCLS imaging |
| **8** | M | Asian | 50 | Stroke | DCD | Asthma, Hypertension | no | PCLS flow |
| **9** | M | White | 46 | Suicide | DCD | None | yes | PCLS flow, SCS |
| **10** | F | Asian | 56 | Stroke | DCD | Hypertension | no | PCLS imaging |
| **11** | F | White | 57 | Stroke | DBD | None | no | PCLS flow, PCLS imaging |
| **12** | M | Hispanic | 27 | Head Trauma | DBD | Asthma | no | PCLS SCS |
| **13** | M | White | 43 | Anoxia | DCD | None | no | BAL, PCLS flow, PCLS imaging |
| **14** | F | Hispanic | 44 | Stroke | DBD | None | no | PCLS imaging, PCLS SCS |
| **15** | M | White | 43 | Anoxia | DCD | None | no | PCLS flow |
| **16** | M | Hispanic | 57 | Stroke | DBD | None | no | BAL |
| **17** | M | White | 50 | Head Trauma | DCD | Hypertension | no | BAL |
| **18** | M | White | 31 | Head Trauma | DCD | None | no | BAL |
| **19** | M | Hispanic | 50 | Stroke | DBD | Hypertension | no | BAL |
| **20** | F | White | 62 | Head Trauma | DBD | Cancer | no | BAL |
| **21** | M | White | 27 | Anoxia | DBD | None | no | BAL |
| **22** | F | Hispanic | 39 | Head trauma | DBD | None | no | BAL |
| **23** | F | White | 58 | Anoxia | DCD | Diabetes, Hypertension | no | BAL |
| **24** | F | White | 62 | Anoxia | DCD | COPD, Hypertension, Hypersensitivity Lung Disease, and Diabetes | yes | BAL |
| **25** | M | White | 32 | Anoxia | DCD | Asthma | no | BAL |
| **26** | M | Hispanic | 25 | Anoxia | DCD | None | no | BAL |
| **27** | M | Hispanic | 33 | Head trauma | DBD | Hypertension | no | BAL |
| **28** | M | White | 33 | Anoxia | DBD | Diabetes | yes | BAL |
| **29** | M | White | 46 | Stroke | DBD | Asthma | no | BAL |
| **30** | M | Asian | 51 | Stroke | DBD | Diabetes, Hypertension | no | BAL |
| **31** | M | White | 35 | Head trauma | DBD | None | no | BAL |
| **32** | M | White | 31 | Anoxia | DBD | None | no | BAL |

| | | | | | | | | |
|---|---|---|---|---|---|---|---|---|
| **33** | M | White | 64 | Anoxia | DCD | Asthma | no | BAL |
| **34** | M | White | 66 | Anoxia | DBD | Hypertension | no | BAL |
| **35** | F | White | 37 | Stroke | DBD | Hypertension | no | BAL |
| **36** | M | White | 28 | Head trauma | DBD | None | no | BAL |
| **37** | M | Black | 22 | Head trauma | DBD | Hypertension | no | BAL |
| **38** | F | White | 27 | Anoxia | DBD | None | no | BAL |
| **39** | F | Hispanic | 26 | Head trauma | DBD | None | no | BAL |
| **40** | F | Hispanic | 57 | Stroke | DBD | Hypertension | yes | BAL |
| **41** | F | Black | 58 | stroke | DBD | Hypertension | no | BAL |
| **42** | F | White | 70 | Stroke | DBD | None | yes | BAL |
| **43** | M | Hispanic | 36 | Anoxia | DBD | None | no | BAL |
| **44** | M | Hispanic | 28 | Anoxia | DCD | None | yes | BAL |
| **45** | F | White | 33 | Anoxia | DCD | None | no | PCLS, BAL |
| **46** | M | Hispanic | 63 | Natural causes | DCD | Diabetes, Hypertension | no | PCLS, BAL |
| **47** | M | Hispanic | 34 | Anoxia | DBD | Diabetes | no | PCLS, BAL |

DBD: donation after brainstem death
DCD: donation after cardiac death
COPD: chronic obstructive pulmonary disease
CAD: coronary artery disease
BAL: bronchoalveolar lavage
PCLS flow: precision cut lung slice flow cytometry
PCLS Imaging: precision cut lung slice immunofluorescence imaging
SCS: single-cell RNA sequencing

## Table S2. COVID-19 subject (endotracheal aspirate samples) demographics.

| Subject | Age | Gender | Ethnicity | ARDS (Y/N) Berlin definition | ETA sampling after intubation (days) | P/F ratio at time of ETT sampling | ICU LOS (days) | Hospital LOS (days) | Death (Y/N) |
|---|---|---|---|---|---|---|---|---|---|
| 1 (1) | 34 | F | White | Yes | 0 | 180 | 12 | 14 | No |
| 2 (365) | 55 | M | White | Yes | 40 | 110 | 35 | 60 | No |
| 3 (414) | 58 | M | Other / Multiple Races | Yes | 1, 3 | 165 | 19 | 27 | No |
| 4 (415) | 62 | F | Black / African American | Yes | 2 | 124 | 14 | 21 | No |
| 5 (419) | 68 | M | Other / Multiple Races | Yes | 7 | 175 | 26 | 50 | No |
| 6 (476) | 59 | M | White | Yes | 12 | 160 | 44 | 78 | No |
| 7 (389) | 66 | F | White | Yes | 2 | 144 | 39 | 55 | No |

ARDS: acute respiratory distress syndrome
ETA: endotracheal tube aspirate
P/F ratio: PaO2/FIO2
ICU LOS: intensive care unit length of stay
Hospital LOS: hospital length of stay

**Table S3. Published studies on ACE2 expression in alveolar macrophages.**

| Reference | Detection of ACE2 in alveolar macrophages | Method |
|---|---|---|
| (*14*) | No | Sc Seq |
| (*25*) | No | Sc Seq / WB |
| (*29*) | Yes | Flow / IHC |
| (*36*) | Yes | IHC |
| (*37*) | No | Sc Seq |
| (*38*) | Yes | IHC |
| (*39*) | Yes | IHC |
| (*40*) | Yes | IHC |
| (*41*) | No | Sc Seq |
| (*42*) | Yes | IHC |
| (*43*) | Yes | IHC |
| (*44*) | No | Sc Seq |
| (*45*) | Yes | Flow |

IHC: immunohistochemistry, Sc Seq: single-cell RNA sequencing, Flow: Flow cytometry, WB: Western Blot

**Table S4. Flow cytometry panel for PCLS experiments.**

| Staining | Antibody (clone) | Lot number | Dye | Catalog number (Supplier) |
|---|---|---|---|---|
| Surface Staining | CD169 | 1621398 | AF594 | FAB5197T (R&D Systems) |
| | ACE2 | BJ07067522 | PE | bs-1004R (BIOSS) |
| | EpCAM | B250368 | BV650 | 324226 (BioLegend) |
| | CD31 (WM59) | 8232937 | BV605 | 562855 (BD Biosciences) |
| | CD45 (HI30) | B333796 | BV421 | 304032 (BioLegend) |
| | CD14 (M5E2) | B275828 | BV711 | 301838 (BioLegend) |
| | CD3 (SK7) | 0314461 | BB700 | 566575 (BD Biosciences) |
| | CD19 (SJ25C1) | 1069967 | BB700 | 566396 (BD Biosciences) |
| | Zombie (viability) | B331984 | NIR | 77184 (BioLegend) |
| | HLA-DR (G46-6) | 1266420 | BUV395 | 564040 (BD Biosciences) |
| Intracellular Staining | Spike | 1619059 | AF647 | FAB105805R (R&D Systems) |
| | dsRNA (J2) | J2-2007 | AF488 | 10010 (Scicons) |

**Table S5. Flow cytometry panel for BAL experiments.**

| Staining | Antibody (Clone) | Lot number | Dye | Reference |
|---|---|---|---|---|
| Surface Staining | CD169 (#908102) | 1621398<br>1686194 | AF594<br>AF647 | FAB5197T (R&D Systems)<br>FAB5197R (R&D Systems) |
| | ACE2 (#171607) | 1623179 | AF594 | FAB9333T (R&D Systems) |
| | ACE2 (#36) | HR15SE2903 | PE | 10108-MM36-P (SinoBiological) |
| | ACE2 (Polyclonal) | HOK0620051 | - | AF933 (R&D Systems) |
| | ACE2 (AC18F) | 0610040-1 | - | 30582 (Cayman Chemical) |
| | CD15 (W6D3) | 1089776 | BV786 | 741013 (BD Biosciences) |
| | CD16 (3G8) | B321940 | BV605 | 302040 (BioLegend) |
| | CD45 (HI30) | B333796 | BV421 | 304032 (BioLegend) |
| | CD14 (M5E2) | B275828 | BV711 | 301838 (BioLegend) |
| | CD3 (SK7) | 0314461 | BB700 | 566575 (BD Biosciences) |
| | CD19 (SJ25C1) | 1069967 | BB700 | 566396 (BD Biosciences) |
| | Zombie (viability) | B331984 | NIR | 77184 (BioLegend) |
| | LiveDead (viability) | 2161888 | PO | L34959 (Invitrogen) |
| | HLA-DR (G46-6) | 1266420 | BUV395 | 564040 (BD Biosciences) |
| | Annexin V | 5190517152 | FITC | 130-092-52 (Miltenyi) |
| | | 51-65875X | PE | 556422 (BD Biosciences) |
| Intracellular Staining | Spike | 1619059 | AF647 | FAB105805R (R&D Systems) |
| | IFITM3 (EPR5242) | GR3416716-1 | AF488 | Ab198559 (Abcam) |
| | IAV NP | VF3017701A | D67J | MA1-7322 (Invitrogen) |

Figure S8D

Antibody: ACE2 (sn0754)

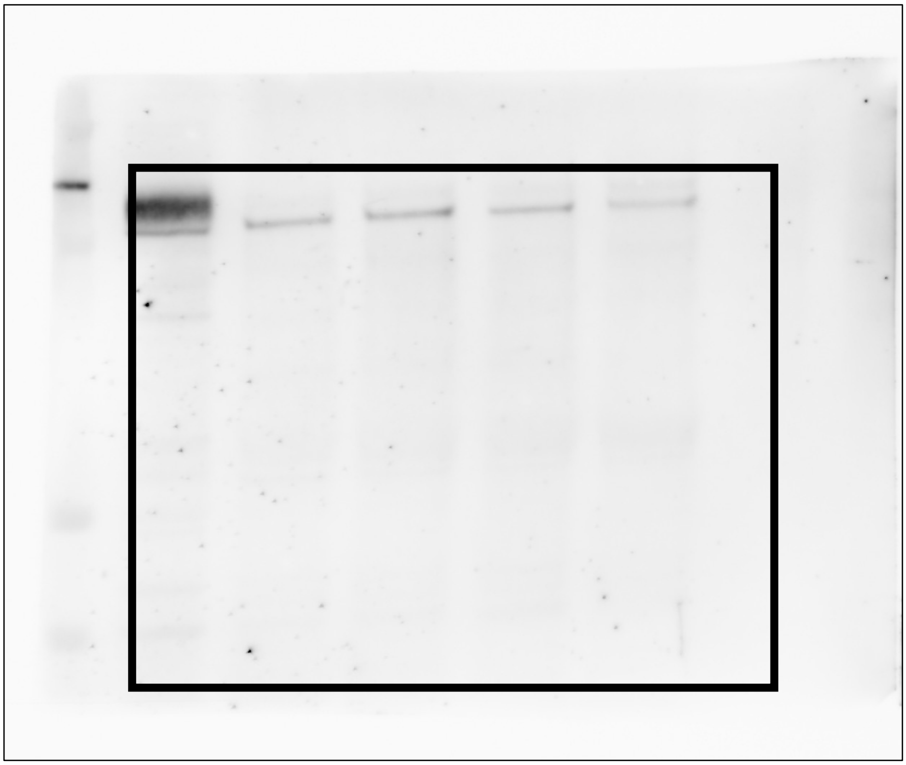

Antibody: GAPDH (PLA0125-100UL)

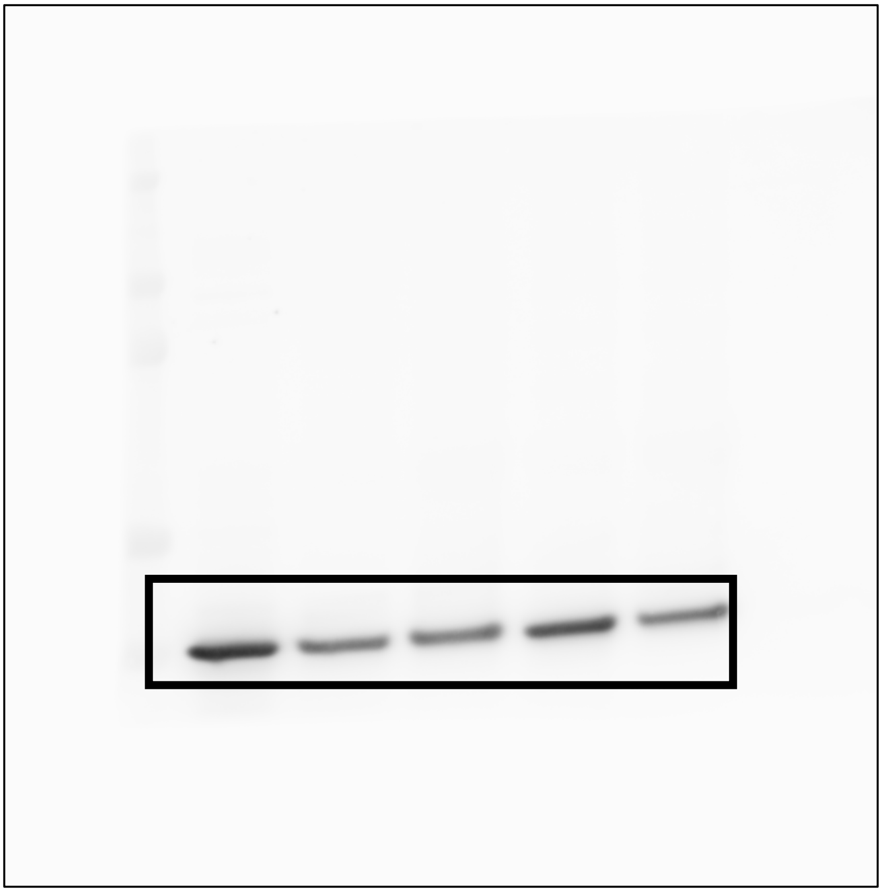

Figure S8E

Antibody: ACE2 (AC18F)

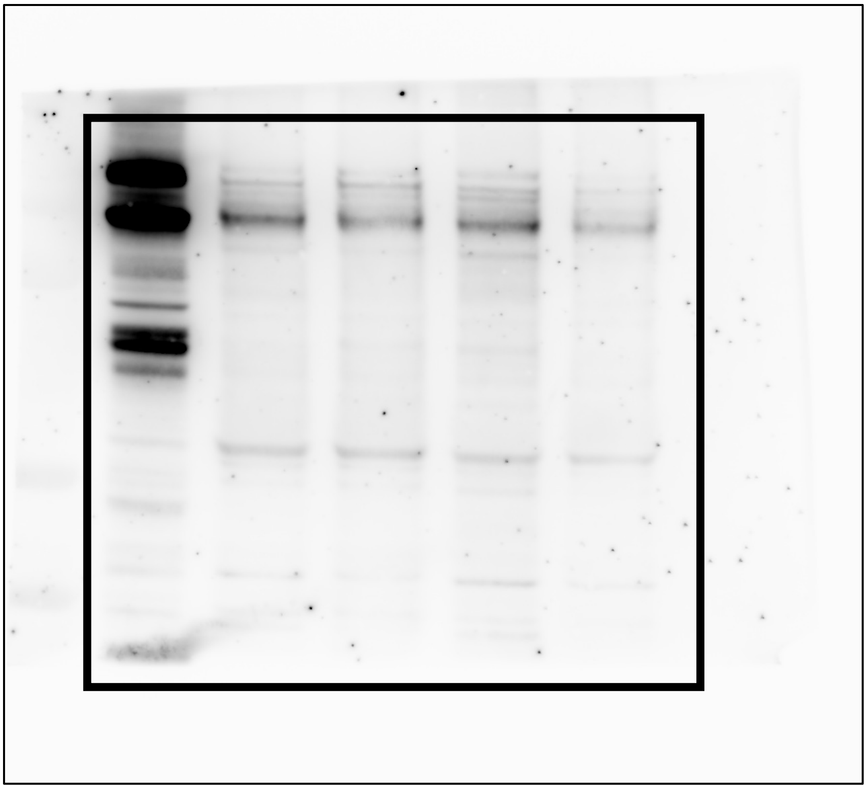

Antibody: GAPDH (PLA0125-100UL)

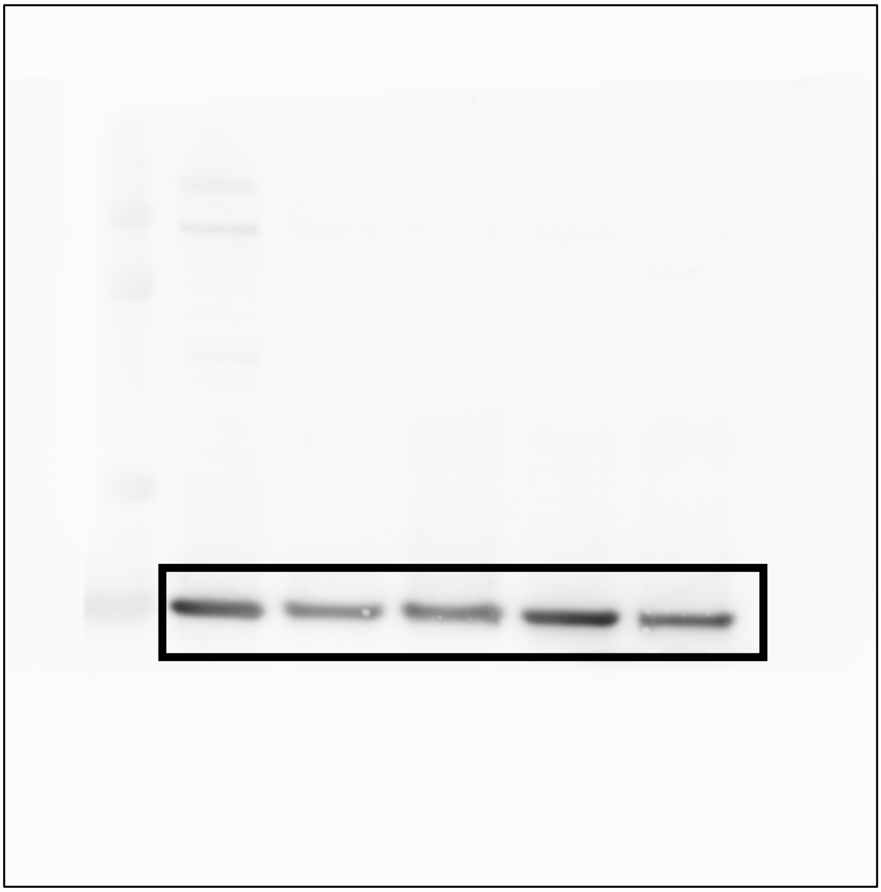

**REFERENCES AND NOTES**

1. M. M. Lamers, B. L. Haagmans, SARS-CoV-2 pathogenesis. *Nat. Rev. Microbiol.* **20**, 270–284 (2022).

2. M. E. Ortiz, A. Thurman, A. A. Pezzulo, M. R. Leidinger, J. A. Klesney-Tait, P. H. Karp, P. Tan, C. Wohlford-Lenane, P. B. McCray, Jr., D. K. Meyerholz, Heterogeneous expression of the SARS-Coronavirus-2 receptor ACE2 in the human respiratory tract. *EBioMedicine* **60**, 102976 (2020).

3. Y. Zhao, Z. Zhao, Y. Wang, Y. Zhou, Y. Ma, W. Zuo, Single-cell RNA expression profiling of ACE2, the receptor of SARS-CoV-2. *Am. J. Respir. Crit. Care Med.* **202**, 756–759 (2020).

4. A. J. Combes, T. Courau, N. F. Kuhn, K. H. Hu, A. Ray, W. S. Chen, N. W. Chew, S. J. Cleary, D. Kushnoor, G. C. Reeder, A. Shen, J. Tsui, K. J. Hiam-Galvez, P. Munoz-Sandoval, W. S. Zhu, D. S. Lee, Y. Sun, R. You, M. Magnen, L. Rodriguez, K. W. Im, N. K. Serwas, A. Leligdowicz, C. R. Zamecnik, R. P. Loudermilk, M. R. Wilson, C. J. Ye, G. K. Fragiadakis, M. R. Looney, V. Chan, A. Ward, S. Carrillo, U. C. Consortium, M. Matthay, D. J. Erle, P. G. Woodruff, C. Langelier, K. Kangelaris, C. M. Hendrickson, C. Calfee, A. A. Rao, M. F. Krummel, Global absence and targeting of protective immune states in severe COVID-19. *Nature* **591**, 124–130 (2021).

5. D. Mathew, J. R. Giles, A. E. Baxter, D. A. Oldridge, A. R. Greenplate, J. E. Wu, C. Alanio, L. Kuri-Cervantes, M. B. Pampena, K. D'Andrea, S. Manne, Z. Chen, Y. J. Huang, J. P. Reilly, A. R. Weisman, C. A. G. Ittner, O. Kuthuru, J. Dougherty, K. Nzingha, N. Han, J. Kim, A. Pattekar, E. C. Goodwin, E. M. Anderson, M. E. Weirick, S. Gouma, C. P. Arevalo, M. J. Bolton, F. Chen, S. F. Lacey, H. Ramage, S. Cherry, S. E. Hensley, S. A. Apostolidis, A. C. Huang, L. A. Vella; UPenn COVID Processing Unit; M. R. Betts, N. J. Meyer, E. J. Wherry, Deep immune profiling of COVID-19 patients reveals distinct immunotypes with therapeutic implications. *Science* **369**, eabc8511 (2020).

6. L. B. Rodda, J. Netland, L. Shehata, K. B. Pruner, P. A. Morawski, C. D. Thouvenel, K. K. Takehara, J. Eggenberger, E. A. Hemann, H. R. Waterman, M. L. Fahning, Y. Chen, M. Hale, J.

Rathe, C. Stokes, S. Wrenn, B. Fiala, L. Carter, J. A. Hamerman, N. P. King, M. Gale, Jr., D. J. Campbell, D. J. Rawlings, M. Pepper, Functional SARS-CoV-2-specific immune memory persists after mild COVID-19. *Cell* **184**, 169–183.e17 (2021).

7. C. Junqueira, A. Crespo, S. Ranjbar, L. B. de Lacerda, M. Lewandrowski, J. Ingber, B. Parry, S. Ravid, S. Clark, M. R. Schrimpf, F. Ho, C. Beakes, J. Margolin, N. Russell, K. Kays, J. Boucau, U. Das Adhikari, S. M. Vora, V. Leger, L. Gehrke, L. Henderson, E. Janssen, D. Kwon, C. Sander, J. Abraham, M. B. Goldberg, H. Wu, G. Mehta, S. Bell, A. E. Goldfeld, M. R. Filbin, J. Lieberman, FcγR-mediated SARS-CoV-2 infection of monocytes activates inflammation. *Nature* **606**, 576–584 (2022).

8. D. Hashimoto, A. Chow, C. Noizat, P. Teo, M. B. Beasley, M. Leboeuf, C. D. Becker, P. See, J. Price, D. Lucas, M. Greter, A. Mortha, S. W. Boyer, E. C. Forsberg, M. Tanaka, N. van Rooijen, A. Garcia-Sastre, E. R. Stanley, F. Ginhoux, P. S. Frenette, M. Merad, Tissue-resident macrophages self-maintain locally throughout adult life with minimal contribution from circulating monocytes. *Immunity* **38**, 792–804 (2013).

9. E. E. Thornton, M. F. Krummel, M. R. Looney, Live imaging of the lung. *Curr. Protoc. Cytom.* **Chapter 12**, 12 28 11–12 28 12 (2012).

10. E. E. Thornton, M. R. Looney, O. Bose, D. Sen, D. Sheppard, R. Locksley, X. Huang, M. F. Krummel, Spatiotemporally separated antigen uptake by alveolar dendritic cells and airway presentation to T cells in the lung. *J. Exp. Med.* **209**, 1183–1199 (2012).

11. F. Baharom, G. Rankin, A. Blomberg, A. Smed-Sorensen, Human lung mononuclear phagocytes in health and disease. *Front. Immunol.* **8**, 499 (2017).

12. K. J. Travaglini, A. N. Nabhan, L. Penland, R. Sinha, A. Gillich, R. V. Sit, S. Chang, S. D. Conley, Y. Mori, J. Seita, G. J. Berry, J. B. Shrager, R. J. Metzger, C. S. Kuo, N. Neff, I. L. Weissman, S. R. Quake, M. A. Krasnow, A molecular cell atlas of the human lung from single-cell RNA sequencing. *Nature* **587**, 619–625 (2020).

13. D. F. Boyd, E. K. Allen, A. G. Randolph, X. J. Guo, Y. Weng, C. J. Sanders, R. Bajracharya, N. K. Lee, C. S. Guy, P. Vogel, W. Guan, Y. Li, X. Liu, T. Novak, M. M. Newhams, T. P. Fabrizio, N. Wohlgemuth, P. M. Mourani, P. P. I. C. I. Investigators, T. N. Wight, S. Schultz-Cherry, S. A. Cormier, K. Shaw-Saliba, A. Pekosz, R. E. Rothman, K. F. Chen, Z. Yang, R. J. Webby, N. Zhong, J. C. Crawford, P. G. Thomas, Exuberant fibroblast activity compromises lung function via ADAMTS4. *Nature* **587**, 466–471 (2020).

14. R. A. Grant, L. Morales-Nebreda, N. S. Markov, S. Swaminathan, M. Querrey, E. R. Guzman, D. A. Abbott, H. K. Donnelly, A. Donayre, I. A. Goldberg, Z. M. Klug, N. Borkowski, Z. Lu, H. Kihshen, Y. Politanska, L. Sichizya, M. Kang, A. Shilatifard, C. Qi, J. W. Lomasney, A. C. Argento, J. M. Kruser, E. S. Malsin, C. O. Pickens, S. B. Smith, J. M. Walter, A. E. Pawlowski, D. Schneider, P. Nannapaneni, H. Abdala-Valencia, A. Bharat, C. J. Gottardi, G. R. S. Budinger, A. V. Misharin, B. D. Singer, R. G. Wunderink; NU SCRIPT Study Investigators, Circuits between infected macrophages and T cells in SARS-CoV-2 pneumonia. *Nature* **590**, 635–641 (2021).

15. W. G. Bain, H. F. Penaloza, M. S. Ladinsky, R. van der Geest, M. Sullivan, M. Ross, G. D. Kitsios, B. A. Methe, B. J. McVerry, A. Morris, A. M. Watson, S. C. Watkins, C. M. St Croix, D. B. Stolz, P. J. Bjorkman, J. S. Lee, Lower respiratory tract myeloid cells harbor SARS-Cov-2 and display an inflammatory phenotype. *Chest* **159**, 963–966 (2021).

16. E. Papalexi, R. Satija, Single-cell RNA sequencing to explore immune cell heterogeneity. *Nat. Rev. Immunol.* **18**, 35–45 (2018).

17. X. Fang, M. A. Matthay, Measurement of protein permeability and fluid transport of human alveolar epithelial type II cells under pathological conditions. *Methods Mol. Biol.* **1809**, 121–128 (2018).

18. O. M. Gbenedio, C. Bonnans, D. Grun, C. Y. Wang, A. J. Hatch, M. R. Mahoney, D. Barras, M. Matli, Y. Miao, K. C. Garcia, S. Tejpar, M. Delorenzi, A. P. Venook, A. B. Nixon, R. S. Warren, J. P. Roose, P. Depeille, RasGRP1 is a potential biomarker to stratify anti-EGFR therapy response in colorectal cancer. *JCI Insight* **5**, e127552 (2019).

19. A. Bharat, S. M. Bhorade, L. Morales-Nebreda, A. C. McQuattie-Pimentel, S. Soberanes, K. Ridge, M. M. DeCamp, K. K. Mestan, H. Perlman, G. R. Budinger, A. V. Misharin, Flow cytometry reveals similarities between lung macrophages in humans and mice. *Am. J. Respir. Cell Mol. Biol.* **54**, 147–149 (2016).

20. M. Hoffmann, H. Kleine-Weber, S. Schroeder, N. Kruger, T. Herrler, S. Erichsen, T. S. Schiergens, G. Herrler, N. H. Wu, A. Nitsche, M. A. Muller, C. Drosten, S. Pohlmann, SARS-CoV-2 cell entry depends on ACE2 and TMPRSS2 and is blocked by a clinically proven protease Inhibitor. *Cell* **181**, 271–280.e8 (2020).

21. M. A. Schaller, Y. Sharma, Z. Dupee, D. Nguyen, J. Uruena, R. Smolchek, J. C. Loeb, T. N. Machuca, J. A. Lednicky, D. J. Odde, R. F. Campbell, W. G. Sawyer, B. Mehrad, Ex vivo SARS-CoV-2 infection of human lung reveals heterogeneous host defense and therapeutic responses. *JCI Insight* **6**, e148003 (2021).

22. H. Chu, J. F. Chan, Y. Wang, T. T. Yuen, Y. Chai, Y. Hou, H. Shuai, D. Yang, B. Hu, X. Huang, X. Zhang, J. P. Cai, J. Zhou, S. Yuan, K. H. Kok, K. K.-W. To, I. H. Chan, A. J. Zhang, K. Y. Sit, W. K. Au, K. Y. Yuen, Comparative replication and immune activation profiles of SARS-CoV-2 and SARS-CoV in human lungs: An ex vivo study with implications for the pathogenesis of COVID-19. *Clin. Infect. Dis.* **71**, 1400–1409 (2020).

23. E. Sefik, R. Qu, C. Junqueira, E. Kaffe, H. Mirza, J. Zhao, J. R. Brewer, A. Han, H. R. Steach, B. Israelow, H. N. Blackburn, S. E. Velazquez, Y. G. Chen, S. Halene, A. Iwasaki, E. Meffre, M. Nussenzweig, J. Lieberman, C. B. Wilen, Y. Kluger, R. A. Flavell, Inflammasome activation in infected macrophages drives COVID-19 pathology. *Nature* **606**, 585–593 (2022).

24. Z. Zhang, R. Penn, W. S. Barclay, E. S. Giotis, Naïve human macrophages are refractory to SARS-CoV-2 infection and exhibit a modest inflammatory response early in infection. *Viruses* **14**, 441 (2022).

25. L. I. Labzin, K. Y. Chew, K. Eschke, X. Wang, T. Esposito, C. J. Stocks, J. Rae, R. Patrick, H. Mostafavi, B. Hill, T. E. Yordanov, C. L. Holley, S. Emming, S. Fritzlar, F. L. Mordant, D. P. Steinfort, K. Subbarao, C. M. Nefzger, A. K. Lagendijk, E. J. Gordon, R. G. Parton, K. R. Short,

S. L. Londrigan, K. Schroder, Macrophage ACE2 is necessary for SARS-CoV-2 replication and subsequent cytokine responses that restrict continued virion release. *Sci. Signal.* **16**, eabq1366 (2023).

26. M. Shemesh, T. E. Aktepe, J. M. Deerain, J. L. McAuley, M. D. Audsley, C. T. David, D. F. J. Purcell, V. Urin, R. Hartmann, G. W. Moseley, J. M. Mackenzie, G. Schreiber, D. Harari, SARS-CoV-2 suppresses IFNβ production mediated by NSP1, 5, 6, 15, ORF6 and ORF7b but does not suppress the effects of added interferon. *PLOS Pathog.* **17**, e1009800 (2021).

27. Z. Xu, J. H. Choi, D. L. Dai, J. Luo, R. J. Ladak, Q. Li, Y. Wang, C. Zhang, S. Wiebe, A. C. H. Liu, X. Ran, J. Yang, P. Naeli, A. Garzia, L. Zhou, N. Mahmood, Q. Deng, M. Elaish, R. Lin, L. K. Mahal, T. C. Hobman, J. Pelletier, T. Alain, S. M. Vidal, T. Duchaine, M. T. Mazhab-Jafari, X. Mao, S. M. Jafarnejad, N. Sonenberg, SARS-CoV-2 impairs interferon production via NSP2-induced repression of mRNA translation. *Proc. Natl. Acad. Sci. U.S.A.* **119**, e2204539119 (2022).

28. A. S. Neupane, M. Willson, A. K. Chojnacki, E. S. C. F. Vargas, C. Morehouse, A. Carestia, A. E. Keller, M. Peiseler, A. DiGiandomenico, M. M. Kelly, M. Amrein, C. Jenne, A. Thanabalasuriar, P. Kubes, Patrolling alveolar macrophages conceal bacteria from the immune system to maintain homeostasis. *Cell* **183**, 110–125.e11 (2020).

29. C. Wang, J. Xie, L. Zhao, X. Fei, H. Zhang, Y. Tan, X. Nie, L. Zhou, Z. Liu, Y. Ren, L. Yuan, Y. Zhang, J. Zhang, L. Liang, X. Chen, X. Liu, P. Wang, X. Han, X. Weng, Y. Chen, T. Yu, X. Zhang, J. Cai, R. Chen, Z. L. Shi, X. W. Bian, Alveolar macrophage dysfunction and cytokine storm in the pathogenesis of two severe COVID-19 patients. *EBioMedicine* **57**, 102833 (2020).

30. M. Matrosovich, T. Matrosovich, W. Garten, H. D. Klenk, New low-viscosity overlay medium for viral plaque assays. *Virol. J.* **3**, 63 (2006).

31. R. Brauer, P. Chen, Influenza virus propagation in embryonated chicken eggs. *J. Vis. Exp.* 52421 (2015). 10.3791/52421

32. C. S. McGinnis, D. M. Patterson, J. Winkler, D. N. Conrad, M. Y. Hein, V. Srivastava, J. L. Hu, L. M. Murrow, J. S. Weissman, Z. Werb, E. D. Chow, Z. J. Gartner, MULTI-seq: sample multiplexing for single-cell RNA sequencing using lipid-tagged indices. *Nat. Methods* **16**, 619–626 (2019).

33. T. Stuart, A. Butler, P. Hoffman, C. Hafemeister, E. Papalexi, W. M. Mauck, 3rd, Y. Hao, M. Stoeckius, P. Smibert, R. Satija, Comprehensive integration of single-cell data. *Cell* **177**, 1888–1902.e21 (2019).

34. C. Hafemeister, R. Satija, Normalization and variance stabilization of single-cell RNA-seq data using regularized negative binomial regression. *Genome Biol.* **20**, 296 (2019).

35. A. Sarma, S. A. Christenson, A. Byrne, E. Mick, A. O. Pisco, C. DeVoe, T. Deiss, R. Ghale, B. S. Zha, A. Tsitsiklis, A. Jauregui, F. Moazed, A. M. Detweiler, N. Spottiswoode, P. Sinha, N. Neff, M. Tan, P. H. Serpa, A. Willmore, K. M. Ansel, J. G. Wilson, A. Leligdowicz, E. R. Siegel, M. Sirota, J. L. DeRisi, M. A. Matthay; COMET Consortium; C. M. Hendrickson, K. N. Kangelaris, M. F. Krummel, P. G. Woodruff, D. J. Erle, C. S. Calfee, C. R. Langelier, Tracheal aspirate RNA sequencing identifies distinct immunological features of COVID-19 ARDS. *Nat. Commun.* **12**, 5152 (2021).

36. X. Song, W. Hu, H. Yu, L. Zhao, Y. Zhao, X. Zhao, H. H. Xue, Y. Zhao, Little to no expression of angiotensin-converting enzyme-2 on most human peripheral blood immune cells but highly expressed on tissue macrophages. *Cytometry A* 136–145 (2023). 10.1002/cyto.a.24285

37. K. Honzke, B. Obermayer, C. Mache, D. Fathykova, M. Kessler, S. Dokel, E. Wyler, M. Baumgardt, A. Lowa, K. Hoffmann, P. Graff, J. Schulze, M. Mieth, K. Hellwig, Z. Demir, B. Biere, L. Brunotte, A. Mecate-Zambrano, J. Bushe, M. Dohmen, C. Hinze, S. Elezkurtaj, M. Tonnies, T. T. Bauer, S. Eggeling, H. L. Tran, P. Schneider, J. Neudecker, J. C. Ruckert, K. M. Schmidt-Ott, J. Busch, F. Klauschen, D. Horst, H. Radbruch, J. Radke, F. Heppner, V. M. Corman, D. Niemeyer, M. A. Muller, C. Goffinet, R. Mothes, A. Pascual-Reguant, A. E. Hauser, D. Beule, M. Landthaler, S. Ludwig, N. Suttorp, M. Witzenrath, A. D. Gruber, C. Drosten, L. E. Sander, T. Wolff, S. Hippenstiel, A. C. Hocke, Human lungs show limited permissiveness for

SARS-CoV-2 due to scarce ACE2 levels but virus-induced expansion of inflammatory macrophages. *Eur. Respir. J.* **60**, 2102725 (2022).

38. S. A. Baker, S. Kwok, G. J. Berry, T. J. Montine, Angiotensin-converting enzyme 2 (ACE2) expression increases with age in patients requiring mechanical ventilation. *PLOS ONE* **16**, e0247060 (2021).

39. W. Bao, X. Zhang, Y. Jin, H. Hao, F. Yang, D. Yin, X. Chen, Y. Xue, L. Han, M. Zhang, Factors Associated with the expression of ACE2 in human lung tissue: Pathological evidence from patients with normal $FEV_1$ and $FEV_1$/FVC. *J. Inflamm. Res.* **Volume 14**, 1677–1687 (2021).

40. S. Bertram, I. Glowacka, M. A. Muller, H. Lavender, K. Gnirss, I. Nehlmeier, D. Niemeyer, Y. He, G. Simmons, C. Drosten, E. J. Soilleux, O. Jahn, I. Steffen, S. Pohlmann, Cleavage and activation of the severe acute respiratory syndrome coronavirus spike protein by human airway trypsin-like protease. *J. Virol.* **85**, 13363–13372 (2011).

41. Y. Liu, H. Q. Qu, J. Qu, L. Tian, H. Hakonarson, Expression pattern of the SARS-CoV-2 entry genes ACE2 and TMPRSS2 in the respiratory tract. *Viruses* **12**, 1174 (2020).

42. S. Bertram, A. Heurich, H. Lavender, S. Gierer, S. Danisch, P. Perin, J. M. Lucas, P. S. Nelson, S. Pohlmann, E. J. Soilleux, Influenza and SARS-coronavirus activating proteases TMPRSS2 and HAT are expressed at multiple sites in human respiratory and gastrointestinal tracts. *PLOS ONE* **7**, e35876 (2012).

43. K. Brautigam, S. Reinhard, J. A. Galvan, M. Wartenberg, E. Hewer, C. M. Schurch, Systematic investigation of SARS-CoV-2 receptor protein distribution along viral entry routes in humans. *Respiration* **101**, 610–618 (2022).

44. H. Zhang, M. R. Rostami, P. L. Leopold, J. G. Mezey, S. L. O'Beirne, Y. Strulovici-Barel, R. G. Crystal, Expression of the SARS-CoV-2 ACE2 receptor in the human airway epithelium. *Am. J. Respir. Crit. Care Med.* **202**, 219–229 (2020).

45. C. W. Kuo, P. L. Su, T. H. Huang, C. C. Lin, C. W. Chen, J. S. Tsai, X. M. Liao, T. Y. Chan, C. C. Shieh, Cigarette smoke increases susceptibility of alveolar macrophages to SARS-CoV-2 infection through inducing reactive oxygen species-upregulated angiotensin-converting enzyme 2 expression. *Sci. Rep.* **13**, 7894 (2023).
